# Pyrodiversity and Phylodiversity: Insights on Post‐Fire Forest Communities

**DOI:** 10.1111/ele.70460

**Published:** 2026-10-09

**Authors:** Rebecca C. Wilcox, Becky L. Estes, Brent D. Mishler, Sarah J. Jacobs

**Affiliations:** ^1^ Botany Department California Academy of Sciences, Institute for Biodiversity Science and Sustainability San Francisco California USA; ^2^ USDA Forest Service Pacific Southwest Region, Eldorado National Forest Placerville California USA; ^3^ Department of Integrative Biology University of California Berkeley, University and Jepson Herbaria Berkeley California USA

**Keywords:** community assembly, local endemism, prescribed fire, restoration, spatial phylogenetics, understory plants, wildfire

## Abstract

Fire is a fundamental process that structures community assembly and biodiversity patterns; however, altered disturbance regimes associated with anthropogenic change present a major threat to fire‐adapted systems. Reintroducing fire to increase pyrodiversity (spatiotemporal diversity in fire) has been identified as an important step in restoring fire‐adapted western temperate forests. Here, we utilize phylogenetic and taxonomic metrics to assess how pyrodiversity influences plant community assembly dynamics and diversity patterns in the central Sierra Nevada, California, USA. Compared to fire exclusion, regional taxonomic and phylogenetic diversity generally increased after fire. Further, with increased pyrodiversity, community assembly mechanisms diversified and occurrences of diverse plant lineages and local phylogenetic endemism increased, illustrating how evolutionary history influences responses of plants to pyrodiversity. These results demonstrate the importance of restoring mixed‐severity fire to these systems and how phylogenetic tools can provide a valuable way to understand and monitor restoration impacts.

## Graphical abstract

Phylogenetic and taxonomic metrics can be useful ways to explore how the reintroduction of fire influences plant community assembly and diversity patterns. We leveraged a dataset of plant community samples collected before and after three fire events, pairing them with the first application of spatial phylogenetic methods at local spatiotemporal scales. This study reveals how evolutionary history influences the responses of plants to changes in pyrodiversity.

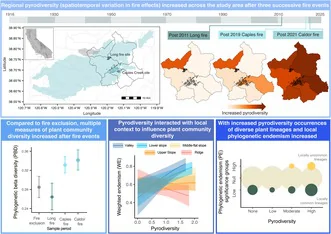

## 1 Introduction

Fire is a fundamental ecological process that structures species traits, community assembly, and biodiversity patterns (He et al. 2019; He and Lamont 2018). Plants in particular have evolved specific traits that allow them to resist, promote, and regenerate after fire (Keeley et al. 2011; Pausas 2019). However, altered disturbance regimes associated with fire suppression, selective management practices and climate change present a major threat to fire‐adapted forest ecosystems (Turner 2010), have severely altered plant community diversity (Tilman and Lehman 2001) and can alter the way that biological communities respond and assemble after fire (Miller and Safford 2020). In response, there has been an effort to consider reintroduceing fire to the landscape and increase pyrodiversity (spatiotemporal variation in fire) using intentional burning (e.g., cultural or prescribed burning) and managed natural ignitions. These types of fires are generally considered to be a critical tools to reduce fuel loads, mimic historic fire regimes and to benefit local plant communities (Stephens et al. 2020). However, despite the importance of plant overstory and understory for multiple aspects of forest health and diversity (Eisenberg et al. 2024; Landuyt et al. 2019), the majority of research generally focuses on how the overstory responds to fire (North et al. 2021), and studies that examine plant community responses are often reactive to suppression wildfires (e.g., large, high‐severity megafires; Abella and Fornwalt 2015; Richter et al. 2019). Therefore, as the number of wild and intentional fires continues to increase, it's important that we explore how plant communities respond to a diversity of fire events and develop holistic metrics to evaluate their response.

Phylogenetics has become a critical tool in community ecology because it improves our understanding of biodiversity patterns and community assembly processes by adding an evolutionary dimension (Webb et al. 2002). Phylogenetic diversity describes the evolutionary breadth represented by species in a community (Faith 1992), and higher phylogenetic diversity has been linked to higher functional diversity, productivity and stability (Srivastava et al. 2012). Spatial phylogenetics considers the distribution of phylogenetic metrics across space and utilizes randomization tests to provide insights into community assembly processes and patterns of phylogenetic diversity and endemism (Mishler 2023). Thus far, studies leveraging spatial phylogenetic approaches have largely focused on examining biogeographic patterns at large spatial scales (Mishler et al. 2014; Thornhill et al. 2016, 2017). However, studying these metrics at finer spatiotemporal scales presents an important opportunity to examine how spatiotemporal variability in disturbances can influence local community assembly mechanisms and patterns of phylogenetic diversity, distinctiveness and local endemism. This paper is the first application of these methods to fine ecological scales.

Pyrodiversity describes the spatiotemporal variability in fire effects (Martin and Sapsis 1992). While current biodiversity has been severely altered and homogenized by fire suppression and more recently by high‐severity wildfires (Perry et al. 2011; Tilman and Lehman 2001), restoring pyrodiversity to be in line with natural fire regimes is hypothesized to increase biodiversity by increasing spatiotemporal variation in forest succession trajectories (and niche diversity) across the landscape (“pyrodiversity‐biodiversity hypothesis”; Martin and Sapsis 1992). However, support for the pyrodiversity‐biodiversity hypothesis is often mixed (Jones and Tingley 2022). To examine how fire impacts plant community diversity, studies have explored regional and local aspects of fire (e.g., pyrodiversity metrics, fire severity) and their influence on plant diversity (Miller and Safford 2020; Wilkin et al. 2021). However, most studies have focused only on taxonomically based diversity metrics (e.g., species richness, beta diversity and Shannon's diversity index; Cohn et al. 2015; Miller and Safford 2020; Weeks et al. 2023). Adding phylogenetic metrics can provide a more nuanced and evolutionarily informed understanding of plant community responses to fire, enabling us to identify how the evolutionary history of plants impacts how community assembly responds to pyrodiversity. For example, the metric relative phylogenetic diversity (RPD) describes the length of phylogenetic branches (relative evolutionary age) present in a plant community (Mishler et al. 2014). If pyrodiversity increases heterogeneity in post‐fire landscapes compared to fire‐excluded landscapes, then we would expect opportunities for distinct groupings of species (e.g., clusters of lineages of relatively young or relatively old evolutionary age) to assemble under different post‐fire conditions.

Community assembly mechanisms affect how species from the regional pool establish at a particular location. Phylogenetic structure can indicate community assembly mechanisms, assuming that closely related species have evolutionarily conserved traits that influence how they respond to their environment (Cavender‐Bares et al. 2004; Webb et al. 2002). There are two deterministic community assembly mechanisms: habitat filtering, where abiotic conditions mean that only species with certain traits can establish and/or persist, resulting in clusters of phylogenetically related species (Webb et al. 2002). For example, moderate‐ to high‐severity fire can create conditions (e.g., high light, low water and low soil nitrogen), where only plants that have certain traits are able to establish (Keeley 1986; Verdú and Pausas 2007). In contrast, competitive exclusion, where local competitive interactions among species with similar traits over time tends to result in the exclusion of some of those species, can lead to distantly related species co‐occurring (termed “phylogenetic overdispersion”; Webb et al. 2002). For example, in systems where there has been a lack of disturbance (i.e., fire exclusion), more time has passed for species to interact, and thus competitive exclusion resulting in distantly related, dissimilar groups of species is more likely (Cavender‐Bares and Reich 2012). Identifying which community assembly mechanisms occur within a community will help us understand the factors that contribute to the observed species composition and diversity of post‐fire communities.

This study took place in the central Sierra Nevada Mountains, California, USA; representative of western temperate forests in that fire has largely been suppressed and few fires have occurred on the landscape in the past century. Between 2011 and 2021 three different moderate‐ to large‐scale, mixed‐severity fires occurred in the region. Moreover, the site was measured for floristic composition before and after each subsequent fire, providing a natural experiment to examine how multiple mixed‐severity fires impact plant communities. Here, we examined a suite of taxonomic and phylogenetic metrics to ask: (1) how does pyrodiversity impact plant community diversity and assembly dynamics across the region? (2) How do fire effects, fire events and local context influence plot‐scale community diversity and assembly dynamics? Addressing these questions will be a critical step towards understanding the importance of evolutionary history in driving species responses to pyrodiversity and will help guide restoration in fire‐adapted communities.

## 2 Material and Methods

### 2.1 Study Site

Our study took place in and around the Caples Creek watershed on the Eldorado National Forest in the central Sierra Nevada (Figure 1b; hereafter the Caples study area; 38°42′38″ N 120°9′9″ W). The study area is on the west slope of the Sierra and is dominated by a broad glaciated valley with granitic rock formations dissected by Caples Creek and the Silver Fork of the American River. The climate is characterized as Mediterranean with warm, dry summers and cool, wet winters (annual average maximum temperature 64.2°F and precipitation 53.6 in; Estes and Gross 2020). The historic pre‐Euro‐American fire‐return interval consisted of low‐severity fires that occurred every 5–35 years (Safford et al. 2011). The study area is similar to other forests in the Sierra; there has been a historic fuel build‐up, tree density has increased and shade‐tolerant tree species have become more dominant. The study area is unique because it is one of the few forested areas in the central Sierra that have not had substantial management (e.g., logging), particularly as the Caples Creek watershed was designated as a Proposed Wilderness in the Eldorado National Forest Plan (Hutchins and Barker 1988). The three different fires in the study area had different ignition sources and therefore different management strategies: the Long, Caples and Caldor fires (Figure 1, Table 1, Supporting Information).

**FIGURE 1:**
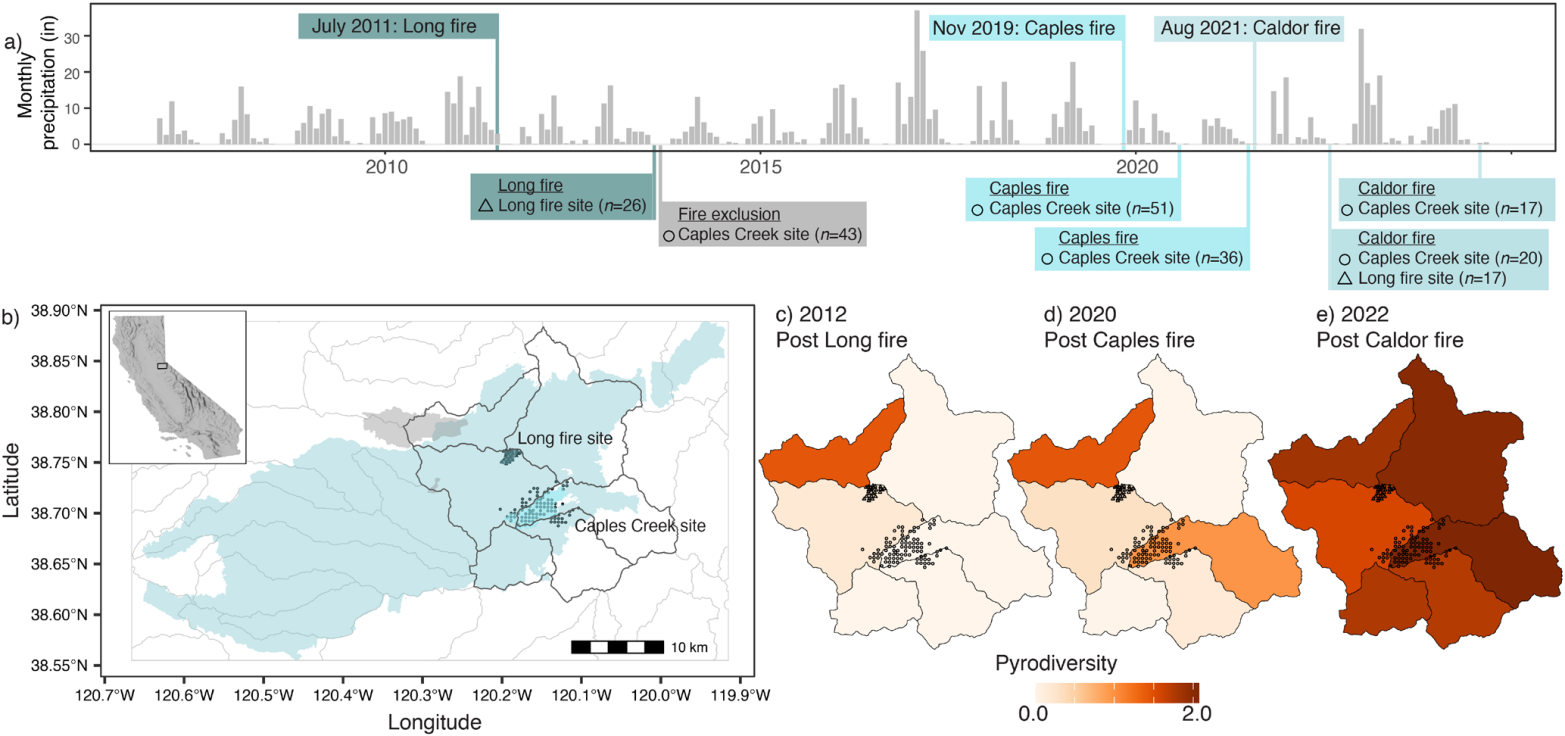
(a) Monthly variation in precipitation (rainfall at the start of each month) for the area (Echo Peak SNOTEL, U.S. Department of Agriculture, National Water and Climate Center n.d.). The flags above the precipitation values indicate the timing of each of the three focal fires (fires that overlap with sampling points), and the flags below indicate when community sampling occurred at each of the two sampling sites. The map (b) shows sub‐watersheds (HUC 12), the Caples Creek study area (sub‐watersheds outlined in black) and the footprints of the fires that have occurred in the study area (colours of the three focal fires match the colours on the flags in the timeline). The study area maps (c–e) show how pyrodiversity has changed across the study area after each fire. (b–e) The symbols represent the two sampling sites (Caples Creek site = circles, Long site = triangles).

**TABLE 1:** Descriptions of the three fires considered in this study.

Fire name	Ignition date–containment date	Size (ha)	Ignition type	Elevation range (m)	Fire management type	Vegetative fire severity^a^	General description
Long	9/12/2011–10/1/2011	202	Lightning	1980–2134	Managed wildfire	Low: 22%	Fire effects were consistent with the natural range of variation for the area
						Low‐moderate: 2%	
						Moderate: 1%	
						High: 0%	
Caples	9/30/2019–10/25/2019	1390	Prescribed fire	1676–2438	Prescribed fire converted to wildfire	Low: 20%	Fire effects were consistent with the natural range of variation for the area (Dailey et al. 2020)
						Low‐moderate: 7%	
						Moderate: 6%	
						High: 33%	
Caldor	8/14/2021–10/21/2021	91,054	Human ignition	548–3048	Suppression wildfire	Low: 21%	As a whole, considered a megafire, but where the fire reburned the Long and Caples footprints fire effects were consistent with natural range of variation for the area
						Low‐moderate: 8%	
						Moderate: 7%	
						High: 46%	

*Note:* Descriptions include ignition date and containment date, size, ignition type, elevation range, fire management type, vegetative fire severity summary and a general description of the fire's effects.

^a^ Derived from the Rapid Assessment of Vegetation Condition after Wildfire (U.S. Department of Agriculture, Forest Service 2026; Table S2) and evaluated across the entirety of the fire's footprint.

### 2.2 Community Sampling

Sampling occurred at two sites: the Caples Creek and the Long fire sites (Figure 1). The sites are similar in their dominant forest types (mixed conifer, red fir), understory plant communities and topographic characteristics (Figure S1). We established plots on a 250‐m grid overlaid on the Caples Creek watershed and the footprint of the Long fire. Across the two sites, we established 115 unique circular fixed‐radius inventory plots, each with a radius of 11.37 m (0.05 ha area; U.S. Department of Agriculture, Forest Service 2019). Plots were surveyed for floristic (identity and abundance) and structural elements (shrub and tree cover). We recorded all taxa within the plot and identified them to at least the genus level. We recorded percent cover to the nearest 1% (abundance) for each plant species in each plot using visual estimates. Sampling occurred from June to August and was conducted by trained botanists. Sampling occurred in four sampling periods. *First*, the post‐Long fire; *second*, the fire exclusion (representing the period of no recorded fires since 1916; Dailey et al. 2020), where plots were sampled in the Caples Creek site before any modern‐era fires occurred; *third*, the post‐Caples fire; and *fourth*, the post‐Caldor fire (Figure 1, Table S1).

### 2.3 Phylogenetic Tree

We used the phylogeny constructed for native California plants by Thornhill et al. (2017). Based on the availability of genetic data, those authors grouped species based into operational taxonomic units (OTUs), sometimes at the genus level, to estimate the phylogeny. Our taxon list included native and non‐native genera, species, subspecies and varieties (*n* = 346). We matched all native taxa (*n* = 331) to the OTUs from the Thornhill et al. (2017) OTU list, while checking for synonyms (Supporting Information). This resulted in a list of 179 OTUs. The Thornhill et al. (2017) tree does not include non‐native taxa; therefore, the 15 non‐native taxa in our list were not included in the phylogenetic analyses.

### 2.4 Diversity Metrics

#### 2.4.1 Alpha Diversity Metrics

We calculated five alpha diversity metrics (Table 2) for each plot during each sample period. This included two taxonomic metrics: taxonomic richness (TR) and weighted endemism (WE), and three phylogenetic metrics: phylogenetic diversity (PD), relative phylogenetic diversity (RPD) and phylogenetic endemism (PE; *canaper* package; Nitta et al. 2023). Note that both WE and PE are quantitative measures of local relative endemism (hereafter, endemism), i.e., the inverse of how many total plots the contained taxa (WE) or lineages (PE) occur in.

**TABLE 2:** Taxonomic and phylogenetic metrics used to describe (a) alpha and (b) beta diversity.

Diversity metrics		Results of statistical tests of alpha phylogenetic metrics
Taxonomic	Phylogenetic	
**(a) Alpha diversity metrics**		
*Taxonomic richness (TR)*—count of all terminal taxa.	*Phylogenetic diversity (PD)*—the total branch length of the minimum path connecting all the terminals to the base of the tree (Faith 1992)	*High*—taxa are more distantly related than expected by chance (phylogenetic overdispersion), which suggests competitive exclusion (Webb et al. 2002).
		*Low* – taxa are more closely related than expected by chance (phylogenetic clustering), which suggests habitat filtering (Webb et al. 2002)
	*Relative phylogenetic diversity (RPD)*—a ratio comparing PD measured on the original tree, with PD measured on a comparison tree that contains the same nodes but with all branches of equal length (Mishler et al. 2014)	*High*—indicates a concentration of relatively long branches (Mishler et al. 2014). In regional studies this suggests evolutionary refugia, containing branches that have been pruned by extinction (Mishler 2023)
		*Low*—indicates a concentration of relatively short branches (Mishler et al. 2014). In regional studies this suggests locations containing branches that have gone through recent diversification (Mishler 2023)
*Weighted endemism (WE)—*species are down‐weighted by the number of plots in which they occur within the study area, and the index is the sum of the species values for each plot (Linder 2001).	*Phylogenetic endemism (PE)—*measure of how range restricted lineages are in a given location this is calculated using a tree whose branch lengths have been divided by the number of plots they occur in (Rosauer et al. 2009)	*High*—indicates a significant concentration of locally uncommon lineages. In regional studies, this indicates centers of endemism (Mishler et al. 2014).
		*Low*—indicates a significant concentration of common lineages
**(b) Beta diversity metrics**		
*Taxonomic beta diversity (TBD)—*measure of variation in species composition between different plots (Anderson et al. 2011).	*Phylogenetic beta diversity (PBD)—*phylogenetic beta diversity (phyloturnover) counts the lengths of the branches that are not shared between two plots (Faith et al. 2009)	

*Note:* Statistical tests performed for alpha PD, RPD and PE can provide insights if taxa within a particular community (plot) have values that are higher or lower than expected by chance (random). Significant PD can provide evidence of potential community assembly mechanisms, while significant results from RPD and PE can reveal clusters of distinct groups of species based on relative branch length and weighted endemism respectively; potential interpretations are given in the third column.

#### 2.4.2 Beta Diversity Metrics

We calculated two beta diversity metrics for each plot during each sample period: taxonomic beta diversity (TBD) and phylogenetic beta diversity (PBD). To calculate dissimilarity matrices, we used Bray–Curtis dissimilarity for TBD (*vegan* package; Oksanen et al. 2025) and phylo‐Sorenson's dissimilarity for PBD (*betapart* package; Baselga et al. 2023). Given that we were interested in evaluating changes in beta diversity across a priori categories (i.e., sample periods), we created one pairwise dissimilarity matrix for each metric that included all plot surveys (initial and resurveys) across the four sampling periods. We then used those matrices and calculated the distance‐to‐centroid values for each plot in each sample period (*vegan* package; Anderson et al. 2011; Oksanen et al. 2025).

For both alpha and beta diversity metrics, we used the full species list (*n* = 346) for taxonomic metrics (TR, WE and TBD) and the OTU list as described above (*n* = 179) for phylogenetic metrics (PD, PE, RPD and PBD).

### 2.5 Fire Effects

We measured fire effects in two ways. First, we considered fire severity because it is a commonly used metric. During each plot visit, we assessed fire severity on a scale of 0–5 (USDI 2003): from zero‐not burned; to five‐very high‐intensity burn, near or total mortality of overstory (Supporting Information; Table S2). Second, we calculated pyrodiversity as the functional dispersion of fire frequency, severity and patch size across a local landscape (*FD* package; Laliberté and Legendre 2010; Steel et al. 2021; Wilkin et al. 2021). We used fire perimeter data from the Fire and Resource Assessment Program (California Department of Forestry and Fire Protection (CAL FIRE) Fire and Resource Assessment Program 2026) to calculate fire frequency. We used fire severity data from the Rapid Assessment of Vegetation Condition after Wildfire (RAVG; U.S. Department of Agriculture, Forest Service 2026) to calculate fire severity and patch size. Together, these datasets contained reliable data for fires (> 90 ha) in the region between 1985 and 2024. We calculated fire return intervals based on the first and final years of the dataset, and the difference between burn years of overlapping fire perimeters. To pair the remote sensing with the plot‐level fire severity scores, we chose to calculate fire severity based on the percent change in canopy cover (five‐class data; Miller et al. 2009; Table S2). We calculated patch size by defining distinct patches in each burn year using the five‐class fire severity levels and calculating the size of each contiguous patch. We used a recency‐weighted averaging approach to calculate the fire regime traits for each pixel (30 × 30m) on the landscape, where more recent fires receive the greatest weight, and subsequent fires decline in weight (Steel et al. 2021; decay rate = 0.5). Functional dispersion values close to zero indicate that fire histories are similar (low pyrodiversity), while higher values indicate more heterogeneity in fire histories (high pyrodiversity).

We calculated pyrodiversity at two scales: first, we calculated functional dispersion of sub‐watersheds after each of the three fires (HUC 12; Figures 1, S3; regional scale). Second, we calculated the functional dispersion of the area around the plot corresponding to the year in which the community was sampled (50, 100, 150, 200 and 250 m buffer; local scale). We performed model selection to determine the most relevant spatial scale (buffer size) for each diversity metric to be used in subsequent analyses (Supporting Information).

### 2.6 Analysis

We used generalized linear mixed models (GLMM) to predict whether diversity metrics differed between each of the four sample periods (regional scale) and whether they are more likely to be impacted by the fire event, fire effects and/or local context (local scale). In all models, we included a random effect of plot number (random intercept) to account for repeated observations at a given plot. We did not have a sufficient number of groups to incorporate site as a random effect (Gelman and Hill 2007); therefore, we included study site as a fixed effect to account for variation attributed to specific sites. In our models, we checked for collinearity among fixed effects and removed terms when appropriate (VIF > 3, Zuur et al. 2010). For each model, we used the appropriate error distribution for that diversity metric (Table S3; *glmmTMB* package; Brooks et al. 2017) and evaluated model fit using marginal and conditional *R* ^2^ values (Nakagawa and Schielzeth 2013; Zuur et al. 2010). All analyses were conducted in the program R version 4.3.3 (R Core Team 2024).

#### 2.6.1 Regional Scale

We constructed GLMMs to examine whether regional patterns of plant community diversity changed across four sample periods (*n* = 210). We constructed seven GLMMs, each with a different diversity metric (Table 2) as the response variable. We included sample period as a fixed effect. To tell how sample periods differed, we ran all models with each of our three reference sample periods: fire exclusion, the Long fire and the Caples fire. Sample periods were considered to be significantly different if the 95% confidence interval around the parameter estimate (*β*) did not overlap zero (Burnham and Anderson 2004; Zuur et al. 2007).

#### 2.6.2 Local Scale

We constructed model sets of GLMMs to examine whether local patterns of post‐fire plant community diversity differed based on fire effects (fire severity analysed as a categorical variable, and pyrodiversity = functional dispersion) and/or local context: the topographic position index (proxy for sun exposure and water availability), solar radiation (measure of sun exposure), elevation or dominant vegetation type (shrub, tree, shrub/tree, tree/shrub; Supporting Information). We subset the data to include only the plots that were measured after a fire event (*n* = 167). We constructed seven model sets, each with one of the diversity metrics as the response variable (Table 2). In each model set, we included 23 models (including single, two‐way and three‐way interactions) with the fixed effects listed above (Table S4). We removed individual models from a set if there were convergence issues. We compared models using AICc model selection, and a factor was considered to have a significant effect if the model ranked highly (< 2 ΔAICc) and had high model weight (Burnham and Anderson 2004).

#### 2.6.3 Statistical Tests of Alpha Phylogenetic Metrics

Because each of the phylodiversity metrics is expected to be generally correlated with TR, given that they are additive measures, we ran randomizations of the dataset that controlled for the number of OTUs, then compared null distributions to the observed results to estimate significant departures from expectations (two‐tailed tests) of PD, RPD and PE (Table 2). The null distributions were created by repeatedly reshuffling the identities of the OTUs found in each plot from the study‐wide occurrence pool, holding the total number of OTUs per plot and the total number of plots per OTU constant (“curveball randomization”, Nitta et al. 2023). The randomizations were generated using the “cpr_rand_test” function using 100,000 replicates and 100,000 iterations (*canaper* package; Nitta et al. 2023). To visualize and interpret the results of the randomization we plotted the same data at both the regional and local scales.

## 3 Results

### 3.1 Regional Scale

After each successive fire event, pyrodiversity increased across the region (Figure 1c–e) and compared to fire exclusion, biodiversity generally increased (Figure 2). Paired taxonomic and phylogenetic alpha and beta diversity metrics were correlated to different degrees (TR:PD, *r* = 0.95; WE:PE, *r* = 0.88; TBD:PBD, *r* = 0.44; Figure S2) and generally showed similar patterns across the four sample periods, with some exceptions. Compared to the fire exclusion sample period, on average, plots had significantly higher TR after all fires (Figure 2a, Tables S5, S6). PD was significantly higher after the Long and Caples fires compared to fire exclusion but did not differ after the Caldor fire (Figure 2b, Tables S5, S6). For relative phylogenetic diversity (RPD), the Caples and Caldor fires did not differ in their levels of RPD, but RPD was significantly lower after the Long fire compared to the other three sample periods (Figure 2c, Tables S5, S6). Weighted endemism (WE) was significantly higher in all post‐fire sample periods compared to fire exclusion, and the Caples and Caldor fires did not significantly differ in their levels of WE (Figure 2d, Tables S5, S6). Conversely, phylogenetic endemism (PE) was similar for fire exclusion and the Caldor fire, but was significantly higher after the Long and Caples fires (Figure 2e, Tables S5, S6). For taxonomic (TBD) and phylogenetic beta diversity (PBD), the Caldor fire had the highest values and fire exclusion and Long fire were similarly low (Figure 2f,g, Tables S5, S6). After the Caples fire, TBD did not differ from fire exclusion; however, PBD was significantly higher (Figure 2f,g, Tables S5, S6).

**FIGURE 2:**
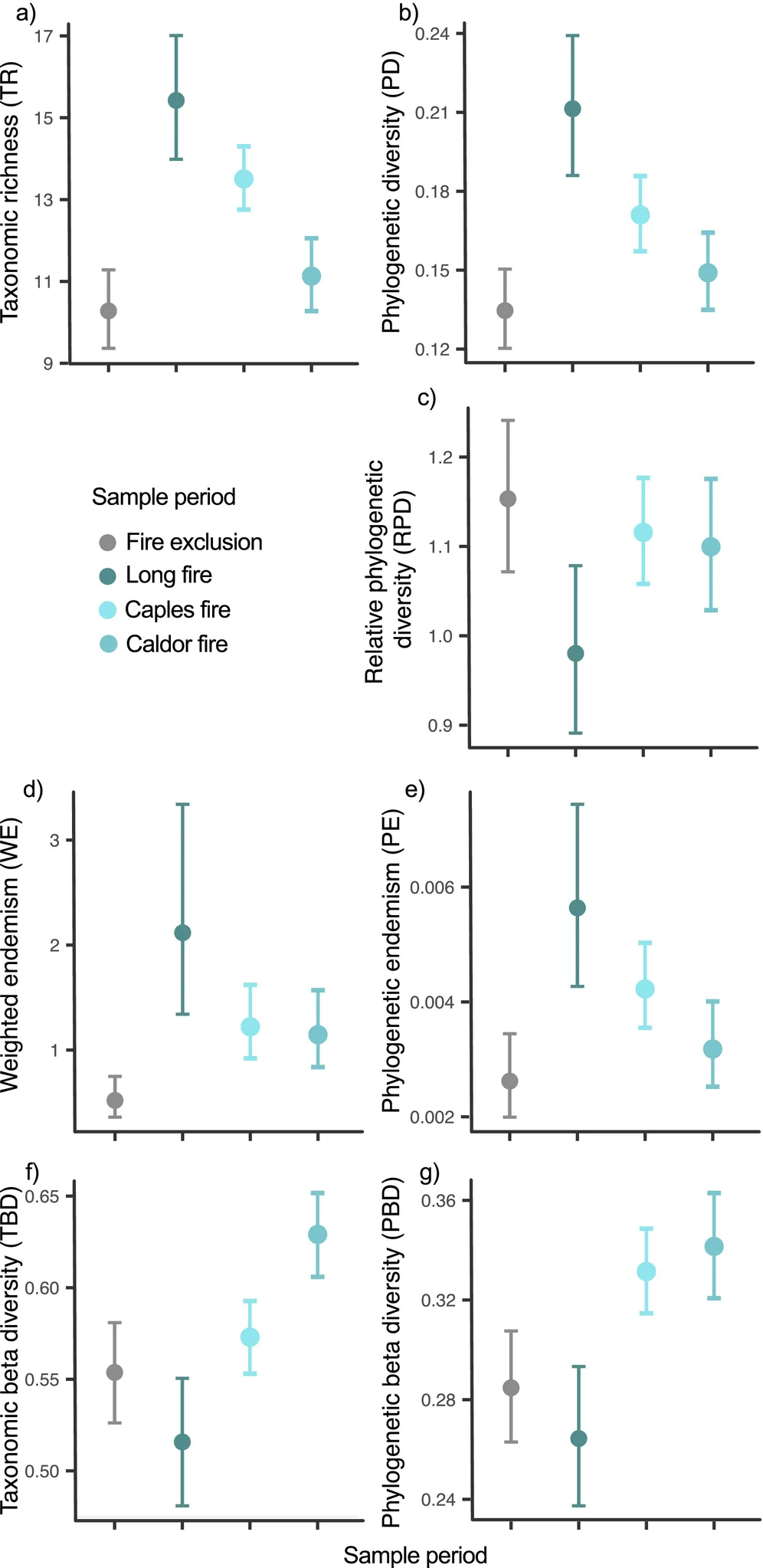
Model estimated relationship between sample period and (a) taxonomic richness (TR), (b) phylogenetic diversity (PD), (c) relative phylogenetic diversity (RPD), (d) weighted endemism (WE), (e) phylogenetic endemism (PE), (f) taxonomic beta diversity (TBD) and (g) phylogenetic beta diversity (PBD). Bars around points indicate 95% confidence intervals.

### 3.2 Local Scale

Community responses to fire were variable across plots and fire events. Plots that burned at low‐ to moderate‐severity for the Long fire (all plots) and Caples fire (plots on canyon bottoms) had growth dominated by shrubs, forbs and graminoids; however, high‐severity fire in the Caples (ridges, where chaparral is dominant) and the Caldor fires (all slope positions, dense tree canopy) resulted in shrub and forb regrowth and shrub, forb and graminoid growth, respectively (Supporting Information; Figures S4–S7).

The spatial scale (buffer size) at which pyrodiversity impacted each diversity metric varied (50–200 m, Table S7). Models including fire severity or pyrodiversity ranked highest for all diversity metrics (Table S8). The model including the interaction between fire event and fire severity ranked highest for TR and PD (Table S8). For both metrics, the Caples and Long fires showed higher values at low‐ to low‐moderate‐fire severity, and the Long fire also showed a peak in diversity at very‐high‐severity fire (Figure 3a,b). In contrast, the Caldor fire showed little difference or a slightly decreasing trend in diversity across fire severity levels (Figure 3a,b). For RPD, the model including fire severity and solar radiation ranked highest (Table S8); on average, plots with low solar radiation had higher RPD at low‐moderate to high burn severities (Figure 3c).

**FIGURE 3:**
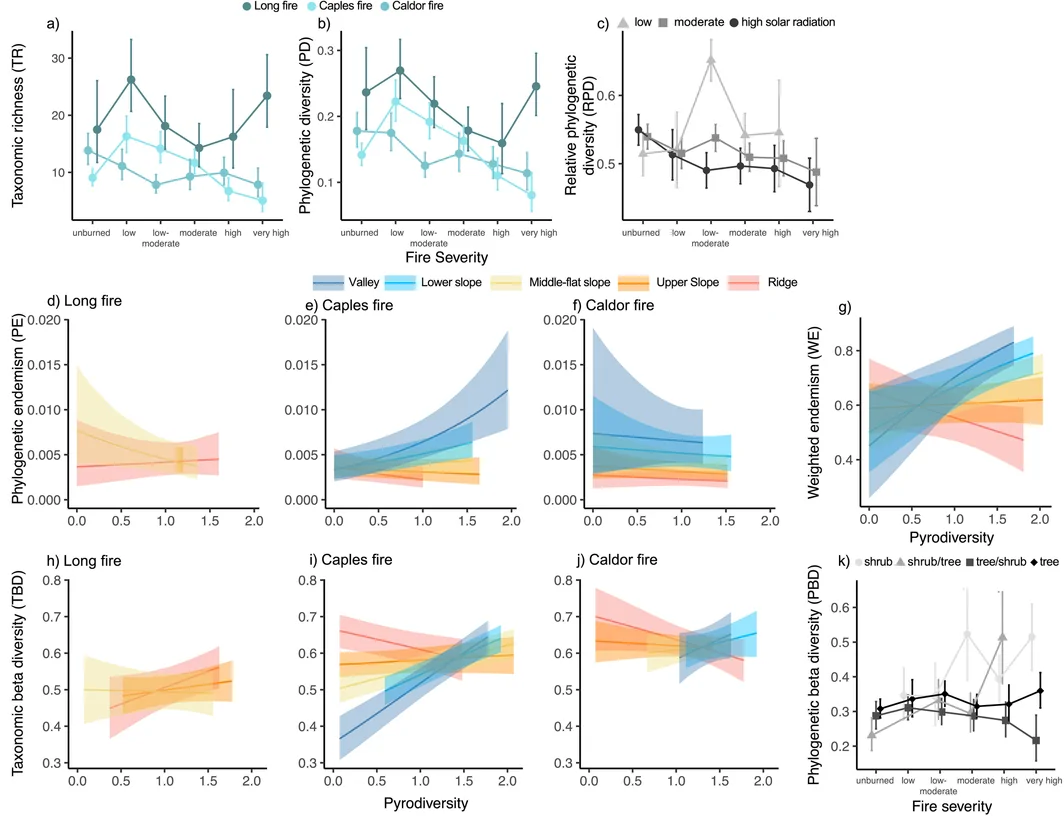
Model estimated relationship between fire severity (zero: Unburned—five: Very high severity) and (a) taxonomic richness (TR), (b) phylogenetic diversity (PD) and (c) relative phylogenetic diversity (RPD) and solar radiation; between pyrodiversity and (d–f) phylogenetic endemism (PE), and (g) weighted endemism (WE), and (h–j) taxonomic beta diversity (TBD) and the topographic position index; and (k) phylogenetic beta diversity (PBD) and vegetation type. For visualization, (c and d–j) solar radiation and topographic position index (both analysed as a continuous factors) were grouped into categories (Supporting Information). Vertical bars around points and ribbons around lines indicate 95% confidence intervals.

The model including the interaction between pyrodiversity and topography ranked highest for WE, and for PE and TBD that relationship differed based on the sample period (Table S8). Endemism and TBD diversity tended to be positively associated with pyrodiversity in valleys, while the relationship shifted to be negative on ridges (Figure 3d–j). However, the patterns deviated after the Long fire for PE and TPD, and after the Caldor fire for PE (Figure 3d,f,h). The model including the interaction between fire severity and dominant vegetation type ranked highest for PBD (Table S8), where PBD tended to be higher for shrub and shrub/tree plots when fire severity was moderate‐ to high‐severity (Figure 3k).

### 3.3 Statistical Tests of Alpha Phylogenetic Metrics

Across all sample periods, fire severity levels, pyrodiversity levels and metrics in the majority of plots did not show a significant pattern (Figure 4). For PD, communities that showed evidence of phylogenetic clustering (significantly low PD) were observed during the fire exclusion, Caples and Caldor sample periods; across plots with low‐moderate‐severity to very high‐severity fire; and across all levels of pyrodiversity (Figure 4a–c). Communities with evidence of phylogenetic overdispersion (significantly high PD) occurred after the Caples and Caldor fires; across plots with low to low‐moderate‐severity fire; and at plots with moderate to high levels of pyrodiversity (Figure 4a–c).

**FIGURE 4:**
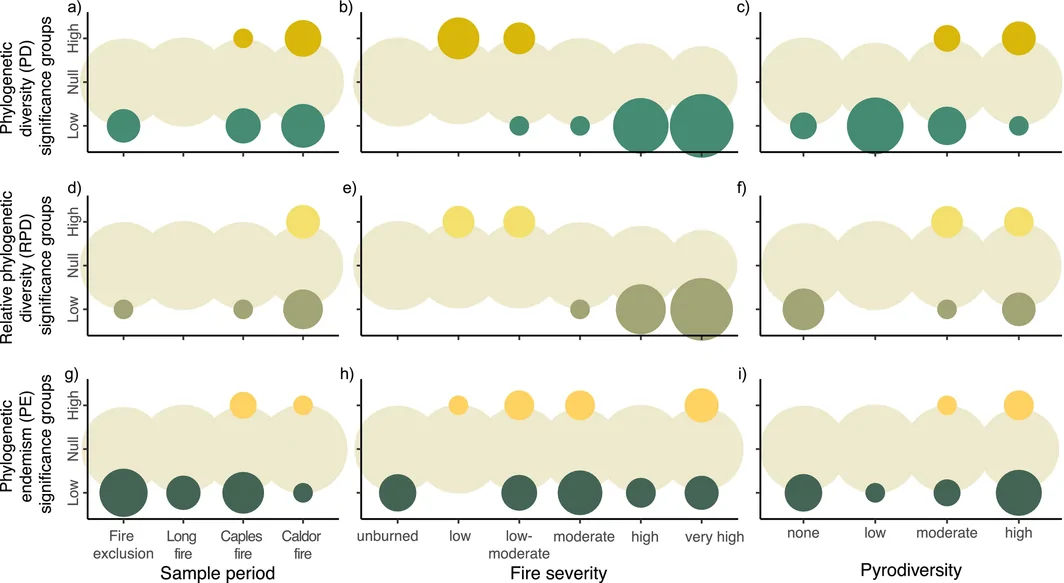
Results from the statistical tests (randomizations) showing the percentage of plots in each significance grouping (null, low or high) for (a–c) phylogenetic diversity (PD), (d–f) relative phylogenetic diversity (RPD) and (g–i) phylogenetic endemism (PE) based on the sample period (column 1), fire‐severity level (column two) and pyrodiversity level (column three). For each plot, the size of the circle refers to the percentage of plots during that sample period, fire‐severity score or pyrodiversity level that were classified at each significance level. Fire‐severity and pyrodiversity figures only include plots that were measured post‐fire. For visualization, pyrodiversity (analysed as a continuous metric) was binned into none, low, moderate and high.

For RPD, communities with evidence of significant concentrations of short branches (significantly low RPD) occurred during the fire exclusion, Caples and Caldor sample periods; across plots with moderate‐ to very high‐severity fire; and at plots with no pyrodiversity or with moderate to high levels of pyrodiversity (Figure 4d–f). Communities with evidence of significant concentrations of long branches (significantly high RPD) occurred after the Caldor fire; across plots with low to low‐moderate‐severity fire; and at plots with moderate to high levels of pyrodiversity (Figure 4d–f).

For PE, all sample periods had plots with concentrations of common branches (significantly low PE); across unburned plots, and plots with low‐moderate‐severity to very high‐severity fire; and at plots with all levels of pyrodiversity (Figure 4g–i). Communities with concentrations of locally endemic branches (significantly high PE) were only observed post‐fire; at plots with low to moderate and very high‐severity fire; and at plots with moderate to high levels of pyrodiversity (Figure 4g–i).

## 4 Discussion

Four key take‐home messages emerged. First, compared to fire exclusion, all regional metrics (except RPD) generally increased after fire events, suggesting that restoring fire to increase pyrodiversity in a system where fire has been excluded for over a century can promote multiple aspects of plant diversity. Second, we observed evidence of significantly high PE (clusters of locally range‐restricted branches) post‐fire and at moderate to high levels of pyrodiversity, suggesting that fire is an important process driving landscape heterogeneity by creating opportunities for endemism to occur via post‐fire succession. Third, topography and dominant vegetation type were important for determining some post‐fire diversity patterns, indicating that local context can interact with recent and historic fire effects to influence post‐fire plant communities. Fourth, in alignment with expectations, we found evidence of habitat filtering (low PD; phylogenetic clustering) in moderate‐ to very‐high‐severity plots; however, counter to expectations, we also found evidence of habitat filtering in fire exclusion plots, suggesting that fire exclusion potentially presents conditions that only some lineages can tolerate.

### 4.1 Restoring Mixed‐Severity Fire

Frequent low‐intensity fires have been an integral part of temperate forest ecosystems for thousands of years, and increasing pyrodiversity via intentional fire is commonly seen as a way to decrease the likelihood of megafires, restore historical fire regimes and promote biodiversity (Stephens et al. 2020). At the regional scale, we found that compared to fire exclusion, multiple diversity metrics generally increased after fire events and subsequent increases in pyrodiversity. This indicates that restoring mixed‐severity fires (within the natural range of variation in the region), even at relatively high frequencies, can promote multiple aspects of plant diversity. At the local scale, diversity metrics varied based on the fire event and/or pyrodiversity, indicating that fire history and local conditions influence how plant communities respond to specific fire events (Miller and Safford 2020) and in turn scale up to influence regional diversity patterns. For example, the Long and Caples fires were the first fires to occur after almost a century of fire exclusion, while the Caldor fire (which, although considered a megafire was actually moderate within the study area; Table 1) burned over the footprints of the other two fires, and fuel loads were likely different. Further, the precipitation conditions before and after the three fires were different, which can affect characteristics of the fires themselves and the growth of plant communities post‐fire (Richter et al. 2019). Therefore, while our results complement some other studies showing that increased pyrodiversity can generally be beneficial for plant diversity (Steel et al. 2025; Wilkin et al. 2021), evidence can be mixed (Jones and Tingley 2022) and practitioners should consider the fire history of their specific landscape when considering the best steps to restore fire regimes.

### 4.2 Pyrodiversity and Endemism

Studies of PE at a local scale have not been done before, and its implications need to be carefully considered. Examining a series of time points on a fine spatial scale allows a nuanced perspective of how spatiotemporally dynamic processes like disturbance regimes create opportunities for relative endemism (clusters of locally range‐restricted branches, high PE) in space and/or time to occur. Here, we observed endemism post‐fire and at moderate‐ to high‐levels of pyrodiversity. This suggests that fire is an important process driving landscape heterogeneity and opportunities for locally uncommon groups of species to occur, which in turn increases biodiversity.

### 4.3 Local Context, Fire and Diversity

One challenge to examining relationships between pyrodiversity and biodiversity is that they can interact at different spatial and temporal scales (Jones and Tingley 2022). For example, in the temperate forests in the western United States, plant (50 m; Wilkin et al. 2021), plant‐pollinator interaction (150 m; Ponisio et al. 2016) and bat (500 m; Steel et al. 2019) richness respond to pyrodiversity at different spatial scales. Here we found that the scale at which pyrodiversity influenced plant diversity differed depending on the metric used (50–200 m) and the direction of the relationship for some metrics changed based on topographic position. This suggests that pyrodiversity has a dynamic relationship with plant communities and local context. Further research could benefit from exploring plant traits (e.g., seed size, dispersal mechanism) and additional local context metrics (e.g., microclimate, soil type) to identify specific mechanisms that cause plant communities to respond to pyrodiversity at different scales. Additionally, continued long‐term monitoring of these sites is needed to understand how pyrodiversity‐biodiversity relationships might change through time (Weeks et al. 2023).

Biophysical gradients can influence vegetation composition and fire severity (Estes et al. 2017). We found that solar radiation interacted with fire severity to influence patterns of relative phylogenetic diversity (RPD; Table 2), a signature of the evolutionary history of plants present in a community. High RPD means the collection of branches present are longer than expected (i.e., a relatively older evolutionary assemblage), while low RPD indicates a collection of shorter branches than expected (i.e., a relatively younger evolutionary assemblage). Specifically, we found that RPD tended to be higher when solar radiation was low and lower when solar radiation was moderate to high. This indicates that communities with relatively longer branches tended to occur in plots on slopes that are wetter and cooler (Northeastern slopes), and that communities with relatively shorter branches occurred on slopes that are drier and hotter (Southwestern slopes).

While, to our knowledge, no one has examined RPD on such a local scale before, the results are comparable with two broader, California‐wide studies. First, Thornhill et al. (2017), found significantly low RPD concentrated in dry parts of the state, although significantly high RPD was scattered out along the precipitation gradient (their Figure 2d). Second, using (non‐phylogenetically informed) biogeographic affinities based on the fossil record (Axelrod 1958; Raven and Axelrod 1978), Stevens et al. (2015) found that the Southern‐xeric taxa (Madro‐Tertiary Geoflora, described as taxa that diversified rapidly with the onset of the Mediterranean climate in California, and thus likely with shorter branches) increased with greater disturbance severity (wildfire and mechanical treatments) and less canopy cover (hotter). While not directly comparable, these results are somewhat in alignment with ours and indicate that more research is warranted to understand how the evolutionary history of the California flora impacts plant community assembly in fire‐adapted landscapes at short‐time scales and local spatial scales.

### 4.4 Community Assembly

Comparing results across a gradient of fire effects provides insights into how fire can influence community assembly patterns. Consistent with expectations, we found that plots that experienced low‐moderate‐severity to very high‐severity fire (especially in higher‐severity fire) were phylogenetically clustered (i.e., close relatives living together), indicating habitat filtering. This likely occurs because in systems where fire is the dominant disturbance, fires alter the environment (e.g., soil characteristics, canopy cover and moisture levels) to create conditions where only species with specific, evolutionarily conserved traits can establish and/or persist (Verdú and Pausas 2007; Dove et al. 2020). Counter to expectations, in the fire exclusion plots, where there has been ample time for species to compete and thus phylogenetic overdispersion might have been expected (Cavender‐Bares and Reich 2012), some plots were phylogenetically clustered instead, indicating that habitat filtering had occurred. This may indicate that long unburned plots might present abiotically harsh conditions (i.e., overshading, increased litter depth and coarse woody debris; Battles et al. 2001; Richter et al. 2019) that restrict many fire‐adapted species from occurring there.

We found evidence of phylogenetic overdispersion (i.e., distant relatives living together) post‐fire, a potential sign of competition excluding close relatives. It is important to note, however, that PD is an imperfect metric for making such inferences if traits are not highly conserved phylogenetically. If traits have evolved fast on the tree, under some circumstances convergent evolution could result in overdispersion (Webb et al. 2002). Therefore, future research should focus on the distribution of traits directly to help interpret phylodiversity patterns and explore how traits are distributed spatially and phylogenetically in western temperate forests (Cavender‐Bares et al. 2009; Larkin et al. 2015; Stevens et al. 2015).

## 5 Conclusions

Our study presents an important first step in applying spatial phylogenetic metrics at fine spatiotemporal scales. In doing so, we elucidate the processes by which pyrodiversity influences biodiversity in post‐fire plant communities: by diversifying community assembly mechanisms and increasing opportunities for diverse plant lineages and temporally and spatially endemic species to occur. Based on our findings, we recommend that practitioners consider increasing pyrodiversity to support plant diversity in Sierra Nevada forests and continue to utilize spatial phylogenetics methods to better understand how evolutionary history influences how plants respond to restoration and impact diversity.

## Author Contributions

All authors contributed to the conceptualization, methodology and investigation of the manuscript. R.C.W. conducted the formal analysis, project administration and visualizations. S.J.J. acquired funding. R.C.W. and B.L.E. curated the data. R.C.W., B.L.E. and S.J.J. supervised the project. R.C.W. wrote the first draft of the manuscript, and all authors contributed substantially to reviewing, editing and revising the manuscript.

## Funding

This work was supported by the California Academy of Sciences, US National Science Foundation, 2325837.

## Supporting information


**Table S1:** The number of plots surveyed during each sampling period at each site.
**Table S2:** Comparison of field (ocular estimates) and remote sensing methods used to evaluate fire severity classes.
**Table S3:** For each diversity metric, the error distribution used in a GLMM for the regional and local analyses.
**Table S4:** List of models included in model sets (GLMM) used to examine whether local patterns of post‐fire plant community diversity differed based on fire severity, pyrodiversity, fire event and/or local context. All models also included study site as a fixed effect and the plot ID as a random effect.
**Table S5:** Sample period parameter estimates from a GLMM examining variation in community diversity between the sample periods. Each cell has a sample periods' parameter estimate and the 95% confidence interval listed in parentheses (lower confidence level, upper confidence level). To tell how sample periods differed from each other we ran models once with “fire exclusion” as the reference sample period, then relevelled the models with “Long fire” as the reference sample period and ran the models again, and then we relevelled the models with “Caples fire” as the reference sample period and ran them one last time. Values in bold indicate a significant difference between the two sample periods (95% confidence interval did not overlap zero).
**Table S6:** R^2^ values from GLMMs examining variation in community diversity between the sample periods (regional analysis). Marginal R^2^ values (R^2^m) consider variation explained by the fixed effects, while conditional R^2^ values (R^2^c) measure variation explained by the fixed and random effects.
**Table S7:** Model selection results assessing the spatial scale at which pyrodiversity has the greatest impact on each biodiversity metric (250, 200, 150, 100 and 50 m). For the phylogenetic endemism model set, we removed “sample site” form the model to reduce complexity, but ultimately removed models when there were issues of singularity. For each diversity metric, models are listed in terms of most supported (highest) to least based on AICc values. Table also includes marginal (R^2^m) and conditional (R^2^c) R^2^ values. The buffer size that ranked highest, was then used in subsequent analyses.
**Table S8:** AICc model selection results evaluating whether local patterns of post‐fire plant community diversity differed based on fire effects (fire severity, pyrodiversity), fire event (the three post fire sample periods) and local context features (dominant vegetation, solar radiation, elevation and topographic position index (TPI)). Models were removed from the set (Table S4) if there were singularity issues, or there was insufficient spread of the data across interacting terms. For each diversity metric (a–g), models are listed in terms of most supported (highest) to least based on AICc values. The table also includes marginal (R^2^m) and conditional (R^2^c) R^2^ values.
**Figure S1:** The (a) Venn diagram (overlap) in taxa (genera) between the Caples creek site (red) and the Long fire sites (blue). The distribution of plots in the two sites by (b) elevation, (c) topographic position and (d) solar radiation.
**Figure S2:** Correlation between each of the paired taxonomic and phylogenetic based metrics including: (a) taxonomic richness (TR) and phylogenetic diversity (PD), (b) weighted endemism (WE) and phylogenetic endemism (PE) and (c) taxonomic beta diversity (TBD) and phylogenetic diversity (PBD) (regional calculation). Pearson's correlation values (r) are listed on the top left of each graph.
**Figure S3:** The spatial distribution of fire return interval (row 1), fire severity (row 2), patch size (row 3) and pyrodiversity (Functional dispersion incorporating the three fire regime metrics) for each sample period (columns), based on sub‐watersheds (HUC12s).
**Figure S4:** Example of a Caples creek plot located on a ridge and South facing, from the fire exclusion sample period (a, sampled 2013) and after the Caples fire (b, sampled 2020).
**Figure S5:** Example of Caples creek plots located in canyon bottom with dense tree cover, from the fire exclusion sample period (a, c; sampled 2013) and after the Caples fire (b, d; sampled 2020).
**Figure S6:** Example of Caples creek plots, representative of all slope positions and with dense canopy cover, from the fire exclusion sample period (a; sampled 2013) and after the Caldor fire (b; sampled 2022).
**Figure S7:** Example of Long plots, representative of high elevation fir forest that burned at low to moderate severity during the Long fire (a; sampled 2013) and during the Caldor fire (b; sampled 2022).
**Figure S8:** Gamma diversity (regional species richness) for each sample period: (grey) fire exclusion, (blue) Long fire, (yellow) Caples fire and (orange) Caldor fire.

## Data Availability

The data and code supporting the results of this manuscript are available on Dryad https://doi.org/10.5061/dryad.m905qfvfs.

## References

[ele70460-bib-0001] Abella, S. R. , and P. J. Fornwalt . 2015. “Ten Years of Vegetation Assembly After a North American Mega Fire.” Global Change Biology 21: 789–802. 10.1111/gcb.12722 25200376

[ele70460-bib-0002] Anderson, M. J. , T. O. Crist , J. M. Chase , et al. 2011. “Navigating the Multiple Meanings of β Diversity: A Roadmap for the Practicing Ecologist: Roadmap for Beta Diversity.” Ecology Letters 14: 19–28. 10.1111/j.1461-0248.2010.01552.x 21070562

[ele70460-bib-0003] Axelrod, D. I. 1958. “Evolution of the Madro‐Tertiary Geoflora.” Botanical Review 24: 433–509.

[ele70460-bib-0004] Baselga, A. , D. Orme , S. Villeger , et al. 2023. “betapart: Partitioning Beta Diversity Into Turnover and Nestedness Components.” R Package Version 1.6.

[ele70460-bib-0005] Battles, J. J. , A. J. Shlisky , R. H. Barrett , R. C. Heald , and B. H. Allen‐Diaz . 2001. “The Effects of Forest Management on Plant Species Diversity in a Sierran Conifer Forest.” Forest Ecology and Management 146: 211–222.

[ele70460-bib-0006] Brooks, M. E. , K. Kristensen , K. J. Van Benthem , et al. 2017. “glmmTMB Balances Speed and Flexibility Among Packages for Zero‐Inflated Generalized Linear Mixed Modeling.” R Journal 9: 378–400.

[ele70460-bib-0007] Burnham, K. P. , and D. R. Anderson . 2004. “Multimodel Inference: Understanding AIC and BIC in Model Selection.” Sociological Methods & Research 33: 2261–2304.

[ele70460-bib-0008] California Department of Forestry and Fire Protection (CAL FIRE) Fire and Resource Assessment Program . 2026. California Historical Wildland Fire Perimeters (Firep25_1). California Open Data Portal.

[ele70460-bib-0009] Cavender‐Bares, J. , D. D. Ackerly , D. A. Baum , and F. A. Bazzaz . 2004. “Phylogenetic Overdispersion in Floridian Oak Communities.” American Naturalist 163: 823–843. 10.1086/386375 15266381

[ele70460-bib-0010] Cavender‐Bares, J. , K. H. Kozak , P. V. A. Fine , and S. W. Kembel . 2009. “The Merging of Community Ecology and Phylogenetic Biology.” Ecology Letters 12: 693–715. 10.1111/j.1461-0248.2009.01314.x 19473217

[ele70460-bib-0011] Cavender‐Bares, J. , and P. B. Reich . 2012. “Shocks to the System: Community Assembly of the Oak Savanna in a 40‐Year Fire Frequency Experiment.” Ecology 93: 3304920.

[ele70460-bib-0012] Cohn, J. S. , J. Di Stefano , F. Christie , G. Cheers , and A. York . 2015. “How Do Heterogeneity in Vegetation Types and Post‐Fire Age‐Classes Contribute to Plant Diversity at the Landscape Scale?” Forest Ecology and Management 346: 22–30.

[ele70460-bib-0013] Dailey, S. , A. Reiner , and C. Ewell . 2020. “2019 Caples Fire, Eldorado National Forest.” First Order Fire Effects. Assessed November 2019. USDA Forest Service, Report.

[ele70460-bib-0014] Dove, N. C. , H. D. Safford , G. N. Bohlman , B. L. Estes , and S. C. Hart . 2020. “High‐Severity Wildfire Leads to Multi‐Decadal Impacts on Soil Biogeochemistry in Mixed‐Conifer Forests.” Ecological Applications 30: e02072. 10.1002/eap.2072 31925848

[ele70460-bib-0015] Eisenberg, C. , S. Prichard , M. P. Nelson , and P. Hessburg . 2024. “Braiding Indigenous and Western Knowledge for Climate‐Adapted Forests: An Ecocultural State of Science Report (Wise Path Forward).”

[ele70460-bib-0016] Estes, B. , and S. Gross . 2020. Eldorado National Forest Climate Change Trend Summary. USDA Forest Service: Region 5 Ecology Program.

[ele70460-bib-0017] Estes, B. L. , E. E. Knapp , C. N. Skinner , J. D. Miller , and H. K. Preisler . 2017. “Factors Influencing Fire Severity Under Moderate Burning Conditions in the Klamath Mountains, Northern California, usa .” Ecosphere 8: 1794.

[ele70460-bib-0018] Faith, D. 1992. “Conservation Evaluation and Phylogenetic Diversity.” Biological Conservation 61.1: 1–10.

[ele70460-bib-0068] Faith, D. P. , C. A. Lozupone , D. Nipperess , and R. Knight . 2009. “The Cladistic Basis for The Phylogenetic Diversity (PD) Measure Links Evolutionary Features to Environmental Gradients and Supports Broad Applications of Microbial Ecology’s “Phylogenetic Beta Diversity” Framework.” International Journal of Molecular Sciences 10: 4723–4741. 10.3390/ijms10114723 PMC2808007 20087461

[ele70460-bib-0019] Gelman, A. , and J. Hill . 2007. Data Analysis Using Regression and Multilevel/Hierarchical Models. Cambridge University Press.

[ele70460-bib-0020] He, T. , and B. B. Lamont . 2018. “Baptism by Fire: The Pivotal Role of Ancient Conflagrations in Evolution of the Earth's Flora.” National Science Review 5: 237–254.

[ele70460-bib-0021] He, T. , B. B. Lamont , and J. G. Pausas . 2019. “Fire as a Key Driver of Earth's Biodiversity.” Biological Reviews 94: 1983–2010. 10.1111/brv.12544 31298472

[ele70460-bib-0022] Hutchins, J. H. , and P. F. Barker . 1988. Eldorado National Forest, Land and Resource Management Plan, 429. USDA Forest Service.

[ele70460-bib-0023] Jones, G. M. , and M. W. Tingley . 2022. “Pyrodiversity and Biodiversity: A History, Synthesis, and Outlook.” Diversity and Distributions 28: 386–403.

[ele70460-bib-0024] Keeley, J. E. 1986. “Resilience of Mediterranean Shrub Communities to Fires.” In Resilience in Mediterranean‐Type Ecosystems, edited by B. Dell , A. J. M. Hopkins , and B. B. Lamont , 95–112. Springer Netherlands.

[ele70460-bib-0025] Keeley, J. E. , J. G. Pausas , P. W. Rundel , W. J. Bond , and R. A. Bradstock . 2011. “Fire as an Evolutionary Pressure Shaping Plant Traits.” Trends in Plant Science 16: 406–411. 10.1016/j.tplants.2011.04.002 21571573

[ele70460-bib-0026] Laliberté, E. , and P. Legendre . 2010. “A Distance‐Based Framework for Measuring Functional Diversity From Multiple Traits.” Ecology 91: 299–305. 10.1890/08-2244.1 20380219

[ele70460-bib-0027] Landuyt, D. , E. De Lombaerde , M. P. Perring , et al. 2019. “The Functional Role of Temperate Forest Understorey Vegetation in a Changing World.” Global Change Biology 25: 3625–3641. 10.1111/gcb.14756 31301199

[ele70460-bib-0028] Larkin, D. J. , A. L. Hipp , J. Kattge , et al. 2015. “Phylogenetic Measures of Plant Communities Show Long‐Term Change and Impacts of Fire Management in Tallgrass Prairie Remnants.” Journal of Applied Ecology 52: 1638–1648.

[ele70460-bib-0029] Linder, H. P. 2001. “Plant Diversity and Endemism in Sub‐Saharan Tropical Africa.” Journal of Biogeography 28: 169–182.

[ele70460-bib-0030] Martin, R. E. , and D. B. Sapsis . 1992. “Fires as Agents of Biodiversity: Pyrodiversity Promotes Biodiversity.” Proceedings of the Symposium on Biodiversity of Northwestern California.

[ele70460-bib-0031] Miller, J. D. , E. E. Knapp , C. H. Key , et al. 2009. “Calibration and Validation of the Relative Differenced Normalized Burn Ratio (RdNBR) to Three Measures of Fire Severity in the Sierra Nevada and Klamath Mountains, California, USA.” Remote Sensing of Environment 113: 645–656.

[ele70460-bib-0032] Miller, J. E. D. , and H. D. Safford . 2020. “Are Plant Community Responses to Wildfire Contingent Upon Historical Disturbance Regimes?” Global Ecology and Biogeography 29: 1621–1633.

[ele70460-bib-0033] Mishler, B. D. 2023. “Spatial Phylogenetics.” Journal of Biogeography 50: 1454–1463.

[ele70460-bib-0034] Mishler, B. D. , N. Knerr , C. E. González‐Orozco , A. H. Thornhill , S. W. Laffan , and J. T. Miller . 2014. “Phylogenetic Measures of Biodiversity and Neo‐ and Paleo‐Endemism in Australian Acacia.” Nature Communications 5: 4473. 10.1038/ncomms5473 25034856

[ele70460-bib-0035] Nakagawa, S. , and H. Schielzeth . 2013. “A General and Simple Method for Obtaining *R* ^2^ From Generalized Linear Mixed‐Effects Models.” Methods in Ecology and Evolution 4: 133–142.

[ele70460-bib-0037] Nitta, J. H. , S. W. Laffan , B. D. Mishler , and W. Iwasaki . 2023. “Canaper: Categorical Analysis of Neo‐ and Paleo‐Endemism in R.” Ecography 2023: e06638.

[ele70460-bib-0038] North, M. P. , R. A. York , B. M. Collins , et al. 2021. “Pyrosilviculture Needed for Landscape Resilience of Dry Western United States Forests.” Journal of Forestry 119: 520–544.

[ele70460-bib-0040] Oksanen, J. , G. Simplson , F. Blanchet , et al. 2025. “vegan: Community Ecology Package.” R Package Version 2.6–10.

[ele70460-bib-0041] Pausas, J. G. 2019. “Generalized Fire Response Strategies in Plants and Animals.” Oikos 128: 147–153.

[ele70460-bib-0042] Perry, D. A. , P. F. Hessburg , C. N. Skinner , et al. 2011. “The Ecology of Mixed Severity Fire Regimes in Washington, Oregon, and Northern California.” Forest Ecology and Management 262: 703–717.

[ele70460-bib-0043] Ponisio, L. C. , K. Wilkin , L. K. M'Gonigle , et al. 2016. “Pyrodiversity Begets Plant–Pollinator Community Diversity.” Global Change Biology 22: 1794–1808. 10.1111/gcb.13236 26929389

[ele70460-bib-0044] R Core Team . 2024. R: A Language and Environment for Statistical Computing. R Foundation for Statistical Computing.

[ele70460-bib-0045] Raven, P. J. , and D. Axelrod . 1978. Origin and Relationships of the California Flora. University of California Press.

[ele70460-bib-0046] Richter, C. , M. Rejmánek , J. E. D. Miller , K. R. Welch , J. Weeks , and H. Safford . 2019. “The Species Diversity × Fire Severity Relationship Is Hump‐Shaped in Semiarid Yellow Pine and Mixed Conifer Forests.” Ecosphere 10: e02882.

[ele70460-bib-0047] Rosauer, D. , S. W. Laffan , M. D. Crisp , S. C. Donnellan , and L. G. Cook . 2009. “Phylogenetic Endemism: A New Approach for Identifying Geographical Concentrations of Evolutionary History.” Molecular Ecology 18: 4061–4072. 10.1111/j.1365-294X.2009.04311.x 19754516

[ele70460-bib-0048] Safford, H. D. , K. van de Water , and D. Schmidt . 2011. California Fire Return Interval Departure (FRID) Map, 2010 Version. USDA Forest Service, Pacific Southwest Region and the Nature Conservancy‐California.

[ele70460-bib-0049] Srivastava, D. S. , M. W. Cadotte , A. A. M. MacDonald , R. G. Marushia , and N. Mirotchnick . 2012. “Phylogenetic Diversity and the Functioning of Ecosystems.” Ecology Letters 15: 637–648. 10.1111/j.1461-0248.2012.01795.x 22583836

[ele70460-bib-0050] Steel, Z. L. , B. Campos , W. F. Frick , R. Burnett , and H. D. Safford . 2019. “The Effects of Wildfire Severity and Pyrodiversity on Bat Occupancy and Diversity in Fire‐Suppressed Forests.” Scientific Reports 9: 16300. 10.1038/s41598-019-52875-2 PMC6895131 31806868

[ele70460-bib-0051] Steel, Z. L. , B. M. Collins , D. B. Sapsis , and S. L. Stephens . 2021. “Quantifying Pyrodiversity and Its Drivers.” Proceedings of the Royal Society B: Biological Sciences 288: 20203202. 10.1098/rspb.2020.3202 PMC8059601 33849322

[ele70460-bib-0052] Steel, Z. L. , A. M. Fogg , R. F. Buzbee , et al. 2025. “Finding Floral and Faunal Species Richness Optima Among Active Fire Regimes.” Conservation Biology 39: e70079. 10.1111/cobi.70079 PMC12658942 40450542

[ele70460-bib-0053] Stephens, S. L. , A. L. Westerling , M. D. Hurteau , M. Z. Peery , C. A. Schultz , and S. Thompson . 2020. “Fire and Climate Change: Conserving Seasonally Dry Forests Is Still Possible.” Frontiers in Ecology and the Environment 18: 354–360.

[ele70460-bib-0054] Stevens, J. T. , H. D. Safford , S. Harrison , and A. M. Latimer . 2015. “Forest Disturbance Accelerates Thermophilization of Understory Plant Communities.” Journal of Ecology 103: 1253–1263.

[ele70460-bib-0055] Thornhill, A. H. , B. G. Baldwin , W. A. Freyman , et al. 2017. “Spatial Phylogenetics of the Native California Flora.” BMC Biology 15: 96. 10.1186/s12915-017-0435-x PMC5658987 29073895

[ele70460-bib-0056] Thornhill, A. H. , B. D. Mishler , N. J. Knerr , et al. 2016. “Continental‐Scale Spatial Phylogenetics of Australian Angiosperms Provides Insights Into Ecology, Evolution and Conservation.” Journal of Biogeography 43: 2085–2098.

[ele70460-bib-0057] Tilman, D. , and C. Lehman . 2001. “Human‐Caused Environmental Change: Impacts on Plant Diversity and Evolution.” Proceedings. National Academy of Sciences. United States of America 98: 5433–5440. 10.1073/pnas.091093198 PMC33230 11344290

[ele70460-bib-0058] Turner, M. G. 2010. “Disturbance and Landscape Dynamics in a Changing World.” Ecology 91: 2833–2849. 10.1890/10-0097.1 21058545

[ele70460-bib-0036] United States, U.S. Department of Agriculture, Natural Resource Conservation Service, National Water and Climate Center . n.d. “Air and Water Database.” Accessed December 5, 2024. https://nwcc‐apps.sc.egov.usda.gov/.

[ele70460-bib-0059] U.S. Department of Agriculture, Forest Service . 2019. Common Stand Exam and Inventory and Monitoring Field Guide. USDA Forest Service. https://www.fs.usda.gov/nrm/fsveg/.

[ele70460-bib-0060] U.S. Department of Agriculture, Forest Service (USDA FS) 2026. Rapid Assessment of Vegetation Condition after Wildfire (RAVG). Geospatial Technology and Applications Center (GTAC). https://burnseverity.cr.usgs.gov/ravg/.

[ele70460-bib-0061] USDI National Park Service . 2003. Fire Monitoring Handbook, 274. Fire Management Program Center, National Interagency Fire Center.

[ele70460-bib-0062] Verdú, M. , and J. G. Pausas . 2007. “Fire Drives Phylogenetic Clustering in Mediterranean Basin Woody Plant Communities.” Journal of Ecology 95: 1316–1323.

[ele70460-bib-0063] Webb, C. O. , D. D. Ackerly , M. A. McPeek , and M. J. Donoghue . 2002. “Phylogenies and Community Ecology.” Annual Review of Ecology and Systematics 33: 475–505.

[ele70460-bib-0064] Weeks, J. , J. E. D. Miller , Z. L. Steel , E. E. Batzer , and H. D. Safford . 2023. “High‐Severity Fire Drives Persistent Floristic Homogenization in Human‐Altered Forests.” Ecosphere 14: e4409.

[ele70460-bib-0065] Wilkin, K. , L. Ponisio , D. L. Fry , B. M. Collins , T. Moody , and S. L. Stephens . 2021. “Drivers of Understory Plant Communities in Sierra Nevada Mixed Conifer Forests With Pyrodiversity.” Fire Ecology 17: 30.

[ele70460-bib-0066] Zuur, A. F. , E. N. Ieno , and C. S. Elphick . 2010. “A Protocol for Data Exploration to Avoid Common Statistical Problems: Data Exploration.” Methods in Ecology and Evolution 1: 3–14.

[ele70460-bib-0067] Zuur, A. F. , E. N. Leno , and G. M. Smith . 2007. Analysing Ecological Data. Springer.

