# Supplementary material for: Pyrodiversity and Phylodiversity: Insights on Post‐Fire Forest Communities
Source: Ecol Lett. 2026 Oct 9;29(10):e70460. doi: 10.1111/ele.70460 (PMC13653068; doi:10.1111/ele.70460)
Supplement: Supplementary file 1 [file ELE-29-0-s001.docx]

**Supporting Information**

**Methods**

*The three fires*

In the Caples study area there were three different fires between 2011-2021 which exhibited different ignition sources and therefore different management strategies (Fig 1, Table 1). The Long fire occurred in 2011 following benign weather conditions from a single tree lightning strike and as a result had fire effects that were fairly consistent with the natural range of variability (Table 1). In 2019 the Caples fire started as a planned prescribed fire, but was declared a wildfire due to an extreme wind event. An assessment of fire severity found that the fire displayed a range of impacts from low- to high-severity that were also fairly consistent with the natural range of variability (Dailey *et al.* 2020; Table 1). The Caldor fire started in 2021 by an unplanned human ignition and was driven by high winds and high fuel loads following a prolonged drought (Fig. 1a). As a result, overall, 46% of the Caldor fire burned at high-severity, but where the fire reburned both the Long and Caples footprints the fire was moderate mixed-severity (Table 1).

*Sampling*

Sampling occurred in four sampling periods. First, post Long fire: after the Long fire, 26 plots were sampled in 2013. Second, fire exclusion (representing the period of no recorded fires since 1916): 43 plots were initially sampled in 2013 in the Caples creek site before any of these modern-era fires occurred. Third, post Caples fire: after the Caples fire, 87 plots were sampled in 2020 and 2021 (41 plots resurveyed, 46 new plots) at the Caples creek site. Fourth, post Caldor fire: after the Caldor fire, 54 plots were surveyed in 2022 and 2024 across both the Caples creek and Long fire sites (Fig. 1, Table S1).

*Phylogenetic tree*

Overall, our taxon list included individuals identified to either the genus, species, subspecies, or variety level (*n*=346). From our list, we created a Caples operational taxonomic unit (OTU) list by matching all species, subspecies, and variety level taxa to the OTUs used in the Thornhill et al. (2017) phylogenetic tree while checking for synonyms (Jepson Flora Project 2025; Tropicos 2025; *n*=179). For individuals in our list identified at the genus level (*n*=65), we matched those taxa to the most common OTU from our Caples OTU list (to control for any genetic diversity within a genus due to spatial distribution) belonging to the same genus. If there was a genus that was not represented at the species, subspecies, or variety level in our taxon list (n=8, 2% of taxon list), then for each genus we identified a likely candidate species (and associated OTU from Thornhill et al. (2017)) by examining which species within that genus has a range that includes the Eldorado National Forest, and who is found in yellow pine forest communities (Munz & Keck 1959). Our taxon list also included 15 nonnative taxa which were not included in the phylogeny or subsequent phylogenetic analyses.

*Sampling effort*

Due to variation in sampling effort across the four different sample periods, we also calculated species accumulation curves to determine if sampling effort influenced observed patterns in alpha diversity (as a proxy for our other regional level metrics as well). We used randomized species accumulation curves (sample-based re-sampling curves - rarefaction) to estimate gamma diversity (region level species richness) for each sample period. We used randomized species accumulation curves rather than raw totals, because they account for uneven sampling across the four sample periods and are a statistical expectation of a species accumulation curve (Gotelli & Colwell 2001). To calculate randomized species accumulation curves we used the ‘specaccum’ function (method=’random’, 1000 permutations) in the *vegan* package (Oksanen *et al.* 2025).

We found that when accounting for sampling effort, gamma diversity (regional diversity) was highest during the Caples fire sampling period, followed by the Caldor fire, and the fire exclusion sample periods (Fig. S8). Gamma diversity was higher after the Long fire compared to the fire exclusion sample period, however, it was not significantly different compared to the other two post fire sample periods (Caples and Caldor, Fig. S8).

*Covariates*

We characterized the local plot context in four ways: dominant vegetation type, solar radiation, elevation (meters), and topographic position index (TPI; De Reu *et al.* 2013). Dominant vegetation type was determined based on the composition of tree and shrub cover (cover of shrubs  >0.5m tall, total overstory canopy cover). If tree cover was greater than 50% and shrubs less than 5% then tree was dominant, if tree less than 5% and shrub greater than 50% then shrub, if these designations were not met, then vegetation cover was assigned tree shrub or shrub tree depending on which had a higher cover type.

For the purposes of visualization (Fig. 3) we converted topographic position index (analyzed as a continuous factor) into: ridge: > +1 standard deviation; upper slope: > 0.5 standard deviation  =< 1 standard deviation; middle slope: >-0.5 standard deviation <0.5 standard deviation, slope > 5 deg; flat slope: >=-0.5 standard deviation <=0.5 standard deviation, slope <= 5 deg; lower slopes: >=-1 standard deviation, <0.5 standard deviation; valleys: <-1.0 standard deviation (De Reu *et al.* 2013). However, due to limited coverage, we grouped middle-flat slope and lower slopes into a ‘middle-flat slope’ category. We also converted solar radiation (analyzed as a continuous factor) into: low (<1575, 33rd percentile), moderate (1575-1673), high (>1673, 66th percentile).

*Fire effects*

*Fire severity* - During each plot visit, we assessed fire severity on a scale of 0-5 based on the fire severity classes (reversed) found in the National Park Service Fire Monitoring Handbook (USDI NPS 2003). Zero-not burned; one-low: lightly burned, no significant overstory mortality, groups of surviving saplings and shrubs; two-low-moderate: lightly burned, most saplings and shrubs are dead; three-moderate: moderately burned, understory mostly burned to ground; four-high: significant overstory kill; and five-very high-intensity burn, near or total mortality of overstory (Table S2).

*Pyrodiversity –* We evaluated pyrodiversity by calculating functional dispersion of three fire effects: fire frequency, fire severity, and patch size (Laliberté & Legendre 2010; Steel *et al.* 2021; Steel 2021; Wilkin *et al.* 2021; Fig. S3). At the local scale we calculated the functional dispersion of the area (buffer) around each sampling location (250m, 200m, 150m, 100m, 50m). We performed model selection to determine the most relevant spatial scale (buffer size) for each diversity metric to be used in subsequent analyses. We constructed seven model sets of generalized linear models (GLMMs), each with one of the diversity metrics (Table 2) as the response variable. In each model set we included models with the following fixed effects: 50m buffer, 100m buffer, 150m buffer, 200m buffer, 250m buffer. In all of the models we also included a fixed effect of site (Long site, Caples creek site) and a random effect of plot ID to account for variation attributed to specific sites and plots respectively. For each model we used the appropriate error distribution for that diversity metric (Table S3), ran models using the *glmmTMB* package (Brooks *et al.* 2017). We compared models using AICc model selection (Burnham & Anderson 2004). Then depending on which model ranked highest (Burnham & Anderson 2004), we included the buffer size from that model, as the pyrodiversity metric in the local scale analysis.

**Results**

*Plant community descriptions*

*High severity Caples fire – Ridge/Chaparral dominant* - The ridges and South facing slopes in the Caples Watershed are dominated by native chaparral species with sporadic conifers (Fig. S4a). These areas burned at moderate to high severity (Fig. S4b) following the 2019 Caples Fire but recovered quickly with shrub and forb regrowth evident the following growing season.

*Low to Moderate Severity Caples Fire – Canyon Bottom and Dense Tree Cover -* The drainages in the Caples Fire were dominated by Sierra Nevada mixed conifer forest type with a high density of shade tolerant species and remnant large diameter trees (Fig. S5a, c). Most of these areas had a long history of fire exclusion but burned at low to moderate severity. The understory had been largely suppressed but more light availability led to considerable shrub, forb and graminoid response (Fig. S5b, d).

*High Severity Caldor Fire – All Slope Positions – Dense Tree Canopy -* Some plots did not burn during the Caples fire, but only burned in the 2021 Caldor Fire. These plots were densely populated with conifers trees with little light in the understory (Fig. S6a). These areas burned at high severity consuming both the overstory trees and understory vegetation (Fig. S6b). The understory had been largely suppressed but more light availability led to considerable shrub, forb and graminoid response.

*Low to Moderate Severity Long Fire – High Elevation Fir -* Plots that burned during the Long Fire were mostly low to moderate severity partly due to the timing of the lightning ignitions. These plots were at moderate to high elevation dominated by red fir and montane associated species (Fig. S7a). These plots reburned in the 2021 Caldor Fire at low to moderate severity (Fig. S7b).

**Tables**

Table S1: The number of plots surveyed during each sampling period at each site.

|  | Sample periods | | | | | |
| --- | --- | --- | --- | --- | --- | --- |
|  | Fire exclusion | Long fire | Caples fire | | Caldor fire | |
| Study site | 2013 | 2013 | 2020 | 2021 | 2022 | 2024 |
| Caples Creek | 43 |  | 51 | 36 | 20 | 17 |
| Long fire |  | 26 |  |  | 17 |  |

Table S2: Comparison of field (ocular estimates) and remote sensing methods used to evaluate fire severity classes.

| Ocular estimate | | |  | Remote sensing | |
| --- | --- | --- | --- | --- | --- |
| Description | Fire severity class | Fire severity label |  | Percentage of canopy cover loss | Fire severity class |
| Not applicable (NA) | NA | NA |  | Not mapped | 0; 6-255 |
| Unburned | 0 | Unburned |  | 0% | 1 |
| Lightly burned, no significant overstory mortality, groups of surviving saplings and shrubs | 1 | Low |  | 0-25% | 2 |
| Lightly burned, most saplings and shrubs are dead | 2 | Low-moderate |  | 25-50% | 3 |
| Moderately burned | 3 | Moderate |  | 50-75% | 4 |
| Significant overstory kill, dead needles retained on trees, understory completely burned | 4 | High |  | 75-100% | 5 |
| Total mortality of overstory, no needles retained on trees, understory completely burned | 5 | Very high |  |  |  |

Table S3: For each diversity metric, the error distribution used in GLMM for the regional and local analyses.

| Response variable | Data type | Distribution |
| --- | --- | --- |
| (a) Alpha diversity metrics |  |  |
| Taxonomic richness (TR) | Count data | Poisson |
| Phylogenetic diversity (PD) | Ranged between 0-1 | Beta |
| Relative phylogenetic diversity (RPD) | Positive continuous | Gamma |
| Weighted endemism (WE) | Positive continuous | Gamma |
| Phylogenetic endemism (PE) | Ranged between 0-1 | Beta |
| (b) Beta diversity metrics |  |  |
| Taxonomic beta diversity (TBD) | Ranged between 0-1 | Beta |
| Phylogenetic beta diversity (PBD) | Ranged between 0-1 | Beta |

Table S4: List of models included in model sets (GLMM) used to examine whether local patterns of post-fire plant community diversity differed based on fire severity, pyrodiversity, fire event, and/or local context. All models also included study site as a fixed effect and plot ID as a random effect.

| Model Number | Fixed effects |
| --- | --- |
| 1 | Fire burn severity |
| 2 | Pyrodiversity |
| 3 | Fire burn severity + fire event + fire burn severity * fire event |
| 4 | Pyrodiversity + fire event + fire burn severity * fire event |
| 5 | Topographic position index + fire burn severity + fire burn severity * topographic position index |
| 6 | Solar radiation + fire burn severity + fire burn severity * solar radiation |
| 7 | Elevation + fire burn severity + fire burn severity * elevation |
| 8 | Dominant vegetation type + fire burn severity + fire burn severity * dominant vegetation type |
| 9 | Topographic position index + pyrodiversity + pyrodiversity * topographic position index |
| 10 | Solar radiation + pyrodviersity + pyrodiversity * solar radiation |
| 11 | Elevation + pyrodiversity + pyrodiversity * elevation |
| 12 | Dominant vegetation type + pyrodiversity + pyrodiversity * dominant vegetation type |
| 13 | Fire burn severity + pyrodiversity |
| 14 | Topographic position index + fire burn severity + fire event + fire burn severity * topographic position index * fire event |
| 15 | Solar radiation + fire burn severity + fire event + fire burn severity * solar radiation * fire event |
| 16 | Elevation + fire burn severity + fire event + fire burn severity * elevation * fire event |
| 17 | Dominant vegetation type + fire burn severity + fire event + fire burn severity * dominant vegetation type * fire event |
| 18 | Topographic position index pyrodiversity + fire event + pyrodiversity * topographic position index * fire event |
| 19 | Solar radiation + pyrodiversity + fire event + pyrodiversity * solar radiation * fire event |
| 20 | Elevation + pyrodiversity + fire event + pyrodiversity * elevation * fire event |
| 21 | Dominant vegetation type + pyrodiversity + fire event pyrodiversity * dominant vegetation type * fire event |
| 22 | Fire burn severity + pyrodiversity + fire event + Fire burn severity * pyrodiversity * fire event |
| 23 | Null model |

Table S5: Sample period parameter estimates from GLMM examining variation in community diversity between the sample periods. Each cell has a sample periods' parameter estimate, and the 95% confidence interval listed in parenthesis (lower confidence level, upper confidence level). To tell how sample periods differed from each other we ran models once with "fire exclusion" as the reference sample period, then releveled the models with “Long fire” as the reference sample period and ran the models again, and then we releveled the models with “Caples fire” as the reference sample period and ran them one last time. Values in bold indicate a significant difference between the two sample periods (95% confidence interval did not overlap zero).

|  | Reference sample period | Long fire | Caples fire | Caldor fire |
| --- | --- | --- | --- | --- |
| Taxonomic richness (TR) | Fire exclusion | **0.83 (0.62, 1.05)** | **0.42 (0.3, 0.54)** | **0.32 (0.18, 0.47)** |
|  | Long fire |  | **-0.41 (-0.61, -0.22)** | **-0.51 (-0.68, -0.34)** |
|  | Caples fire |  |  | -0.1 (-0.21, 0.02) |
| Phylogenetic diversity (PD) | Fire exclusion | **0.54 (0.35, 0.74)** | **0.28 (0.17, 0.39)** | 0.12 (-0.02, 0.25) |
|  | Long fire |  | **-0.26 (-0.43, -0.09)** | **-0.43 (-0.58, -0.27)** |
|  | Caples fire |  |  | **-0.16 (-0.27, -0.06)** |
| Relative phylogenetic diversity (RPD) | Fire exclusion | **-0.16 (-0.28, -0.04)** | -0.03 (-0.11, 0.05) | -0.05 (-0.14, 0.05) |
|  | Long fire |  | **0.13 (0.02, 0.24)** | **0.11 (0.01, 0.22)** |
|  | Caples fire |  |  | -0.01 (-0.09, 0.06) |
| Weighted endemism (WE) | Fire exclusion | **1.4 (0.83, 1.98)** | **0.85 (0.51, 1.2)** | **0.79 (0.37, 1.21)** |
|  | Long fire |  | **-0.55 (-1.07, -0.03)** | **-0.61 (-1.1, -0.12)** |
|  | Caples fire |  |  | -0.06 (-0.41, 0.28) |
| Phylogenetic endemism (PE) | Fire exclusion | **0.77 (0.4, 1.14)** | **0.48 (0.23, 0.73)** | 0.19 (-0.09, 0.47) |
|  | Long fire |  | -0.29 (-0.6, 0.02) | **-0.58 (-0.88, -0.27)** |
|  | Caples fire |  |  | **-0.29 (-0.51, -0.06)** |
| Taxonomic beta diversity (TBD) | Fire exclusion | -0.15 (-0.33, 0.02) | 0.08 (-0.03, 0.19) | **0.31 (0.18, 0.45)** |
|  | Long fire |  | **0.23 (0.08, 0.39)** | **0.47 (0.32, 0.61)** |
|  | Caples fire |  |  | **0.23 (0.13, 0.34)** |
| Phylogenetic beta diversity (PBD) | Fire exclusion | -0.1 (-0.28, 0.08) | **0.22 (0.1, 0.34)** | **0.26 (0.13, 0.4)** |
|  | Long fire |  | **0.32 (0.16, 0.48)** | **0.37 (0.21, 0.53)** |
|  | Caples fire |  |  | 0.05 (-0.06, 0.15) |

Table S6: R^2^ values from GLMMs examining variation in community diversity between the sample periods (regional analysis). Marginal R^2^ values (R^2^_m_) consider variation explained by the fixed effects, while conditional R^2^ values (R^2^_c_) measure variation explained by the fixed and random effects.

| Diversity metric | R^2^_m_ | R^2^_c_ |
| --- | --- | --- |
| Taxonomic richness (TR) | 0.15 | 0.79 |
| Phylogenetic diversity (PD) | 0.11 | 0.81 |
| Relative phylogenetic diversity (RPD) | 0.03 | 0.38 |
| Weighted endemism (WE) | 0.09 | 0.50 |
| Phylogenetic endemism (PE) | 0.08 | 0.57 |
| Taxonomic beta diversity (TBD) | 0.18 | 0.90 |
| Phylogenetic beta diversity (PBD) | 0.17 | 0.62 |

Table S7:  Model selection results assessing the spatial scale at which pyrodiversity has the greatest impact on each biodiversity metric (250, 200, 150, 100, 50m). For the phylogenetic endemism model set, we removed ‘sample site’ form the model to reduce complexity, but ultimately removed models when there were issues of singularity. For each diversity metric, models are listed in terms of most supported (highest) to least based on AICc values. Table also includes marginal (R^2^_m_) and conditional (R^2^_c_) R^2^ values. The buffer size that ranked highest, was then used in subsequent analyses.

|  | Buffer size | df | logLik | AICc | ΔAICc | AICc weight | R^2^_c_ | R^2^_m_ |
| --- | --- | --- | --- | --- | --- | --- | --- | --- |
| Taxonomic richness (TR) | 50 meters | 4 | -556.8 | 1121.8 | 0.00 | 0.31 | 0.78 | 0.01 |
|  | 100 meters | 4 | -557.3 | 1122.9 | 1.13 | 0.18 | 0.78 | 0.00 |
|  | 200 meters | 4 | -557.4 | 1123.0 | 1.18 | 0.17 | 0.78 | 0.00 |
|  | 250 meters | 4 | -557.4 | 1123.0 | 1.23 | 0.17 | 0.78 | 0.00 |
|  | 150 meters | 4 | -557.4 | 1123.0 | 1.23 | 0.17 | 0.78 | 0.00 |
| Phylogenetic diversity (PD) | 150 meters | 5 | 211.3 | -412.2 | 0.00 | 0.26 | 0.62 | 0.01 |
|  | 100 meters | 5 | 211.1 | -411.9 | 0.31 | 0.22 | 0.62 | 0.01 |
|  | 200 meters | 5 | 211.0 | -411.6 | 0.63 | 0.19 | 0.64 | 0.00 |
|  | 250 meters | 5 | 211.0 | -411.6 | 0.64 | 0.19 | 0.64 | 0.00 |
|  | 50 meters | 5 | 210.7 | -411.1 | 1.14 | 0.15 | 0.66 | 0.00 |
| Relative phylogenetic diversity (RPD) | 50 meters | 5 | -26.5 | 63.4 | 0.00 | 0.27 | 0.32 | 0.01 |
|  | 250 meters | 5 | -26.8 | 64.0 | 0.61 | 0.20 | 0.30 | 0.01 |
|  | 200 meters | 5 | -26.9 | 64.2 | 0.75 | 0.19 | 0.30 | 0.01 |
|  | 100 meters | 5 | -27.0 | 64.4 | 0.95 | 0.17 | 0.30 | 0.00 |
|  | 50 meters | 5 | -27.0 | 64.4 | 0.96 | 0.17 | 0.30 | 0.00 |
| Weighted endemism (WE) | 150 meters | 5 | -256.5 | 523.3 | 0.00 | 0.33 | 0.35 | 0.03 |
|  | 100 meters | 5 | -256.7 | 523.8 | 0.48 | 0.26 | 0.36 | 0.02 |
|  | 250 meters | 5 | -257.3 | 525.0 | 1.66 | 0.14 | 0.39 | 0.01 |
|  | 200 meters | 5 | -257.3 | 525.0 | 1.68 | 0.14 | 0.39 | 0.01 |
|  | 50 meters | 5 | -257.5 | 525.3 | 2.03 | 0.12 | 0.40 | 0.01 |
| Phylogenetic endemism (PE) | 50 meters | 4 | 718.4 | -1428.5 | 0.00 | 0.28 | 0.32 | 0.00 |
| Taxonomic beta diversity (TBD) | 150 meters | 5 | 180.3 | -350.2 | 0.00 | 0.36 | 0.78 | 0.18 |
|  | 200 meters | 5 | 180.1 | -349.8 | 0.38 | 0.30 | 0.75 | 0.18 |
|  | 100 meters | 5 | 179.4 | -348.4 | 1.75 | 0.15 | 0.73 | 0.17 |
|  | 250 meters | 5 | 179.0 | -347.7 | 2.45 | 0.11 | 0.74 | 0.16 |
|  | 50 meters | 5 | 178.9 | -347.4 | 2.81 | 0.09 | 0.71 | 0.17 |
| Phylogenetic beta diversity (PBD) | 200 meters | 5 | 221.5 | -432.6 | 0.00 | 0.26 | 0.32 | 0.04 |
|  | 250 meters | 5 | 221.5 | -432.6 | 0.04 | 0.25 | 0.30 | 0.04 |
|  | 150 meters | 5 | 221.4 | -432.3 | 0.28 | 0.22 | 0.32 | 0.04 |
|  | 50 meters | 5 | 221.0 | -431.6 | 1.02 | 0.15 | 0.31 | 0.03 |
|  | 100 meters | 5 | 220.7 | -431.1 | 1.55 | 0.12 | 0.28 | 0.02 |

Table S8: AICc model selection results evaluating whether local patterns of post-fire plant community diversity differed based on fire effects (fire severity, pyrodiversity), fire event (the three post fire sample periods), and local context features (dominant vegetation, solar radiation, elevation, topographic position index (TPI)). Models were removed from the set (Table S4) if there were singularity issues, or there was insufficient spread of the data across interacting terms. For each diversity metric (a–g), models are listed in terms of most supported (highest) to least based on AICc values. The table also includes marginal (R^2^_m_) and conditional (R^2^_c_) R^2^ values.

| Model | df | logLik | AICc | ΔAICc | AICc weight | R^2^_m_ | R^2^_c_ |
| --- | --- | --- | --- | --- | --- | --- | --- |
| (a) Taxonomic richness (TR) |  |  |  |  |  |  |  |
| fire severity + fire event + fire event x fire severity | 20 | -516.2 | 1078.2 | 0.00 | 0.85 | 0.25 | 0.79 |
| pyrodiversity + fire event + fire event x pyrodiversity | 8 | -532.5 | 1081.9 | 3.74 | 0.13 | 0.10 | 0.79 |
| pyrodiversity + fire event + TPI + fire event x pyrodiversity x TPI | 14 | -527.9 | 1086.6 | 8.43 | 0.01 | 0.15 | 0.80 |
| pyrodiversity + fire event + elevation + fire event x pyrodiversity x elevation | 14 | -528.6 | 1088.0 | 9.84 | 0.01 | 0.14 | 0.80 |
| pyrodiversity + fire event + TPI + fire event x pyrodiversity x TPI | 14 | -528.7 | 1088.1 | 9.87 | 0.01 | 0.12 | 0.79 |
| pyrodiversity + fire event + dominant vegetation type + fire event x pyrodiversity x dominant vegetation type | 23 | -523.8 | 1101.3 | 23.05 | 0.00 | 0.18 | 0.79 |
| pyrodiversity + fire severity + fire event + fire severity x pyrodiversity x fire event | 37 | -503.1 | 1102.0 | 23.78 | 0.00 | 0.34 | 0.80 |
| fire severity + fire event + TPI + fire event x fire severity x TPI | 38 | -501.9 | 1103.0 | 24.81 | 0.00 | 0.37 | 0.80 |
| fire severity + fire event + elevation + fire event x fire severity x elevation | 38 | -505.1 | 1109.3 | 31.08 | 0.00 | 0.33 | 0.79 |
| pyrodiversity + TPI + pyrodiversity x TPI | 6 | -551.6 | 1115.7 | 37.53 | 0.00 | 0.07 | 0.78 |
| pyrodiversity + elevation + pyrodiversity x elevation | 6 | -552.1 | 1116.6 | 38.44 | 0.00 | 0.04 | 0.78 |
| fire severity + fire event + TPI + fire event x fire severity x TPI | 38 | -509.9 | 1118.9 | 40.66 | 0.00 | 0.31 | 0.79 |
| burn severity | 8 | -551.1 | 1119.1 | 40.90 | 0.00 | 0.05 | 0.77 |
| null | 3 | -557.4 | 1120.9 | 42.75 | 0.00 | 0.00 | 0.78 |
| pyrodiversity | 4 | -556.8 | 1121.8 | 43.61 | 0.00 | 0.01 | 0.78 |
| pyrodiversity + dominant vegetation type + pyrodiversity x dominant vegetation type | 10 | -551.0 | 1123.5 | 45.29 | 0.00 | 0.06 | 0.80 |
| fire severity + solar radiation + fire severity x solar radiation | 14 | -546.5 | 1123.7 | 45.48 | 0.00 | 0.09 | 0.77 |
| pyrodiversity + solar radiation + pyrodiversity x solar radiation | 6 | -556.6 | 1125.7 | 47.48 | 0.00 | 0.01 | 0.79 |
| fire severity + TPI + fire severity x TPI | 14 | -548.6 | 1127.9 | 49.74 | 0.00 | 0.09 | 0.77 |
| pyrodiversity + fire severity + fire severity x pyrodiversity | 14 | -549.0 | 1128.7 | 50.52 | 0.00 | 0.07 | 0.79 |
| fire severity + elevation + fire severity x elevation | 14 | -549.5 | 1129.8 | 51.58 | 0.00 | 0.07 | 0.77 |
| fire severity + dominant vegetation type + fire severity x dominant vegetation type | 23 | -541.0 | 1135.8 | 57.61 | 0.00 | 0.15 | 0.78 |
| fire severity + fire event + dominant vegetation type + fire event x fire severity x dominant vegetation type | 49 | -501.5 | 1142.9 | 64.72 | 0.00 | 0.37 | 0.80 |
| (b) Phylogenetic diversity (PD) |  |  |  |  |  |  |  |
| fire severity + fire event + fire event x fire severity | 21 | 243.4 | -438.3 | 0.00 | 0.72 | 0.26 | 0.86 |
| pyrodiversity + fire event + TPI + fire event x pyrodiversity x TPI | 15 | 234.6 | -436.0 | 2.31 | 0.23 | 0.20 | 0.82 |
| pyrodiversity + fire event + fire event x pyrodiversity | 9 | 226.1 | -433.1 | 5.26 | 0.05 | 0.10 | 0.82 |
| pyrodiversity + TPI + pyrodiversity x TPI | 7 | 221.2 | -427.7 | 10.65 | 0.00 | 0.16 | 0.65 |
| pyrodiversity + fire event + elevation + fire event x pyrodiversity x elevation | 15 | 228.6 | -424.0 | 14.35 | 0.00 | 0.12 | 0.81 |
| pyrodiversity + fire event + TPI + fire event x pyrodiversity x TPI | 15 | 228.0 | -422.9 | 15.45 | 0.00 | 0.10 | 0.82 |
| pyrodiversity + fire event + dominant vegetation type + fire event x pyrodiversity x dominant vegetation type | 24 | 237.9 | -419.4 | 18.89 | 0.00 | 0.21 | 0.86 |
| burn severity | 9 | 218.2 | -417.3 | 21.05 | 0.00 | 0.09 | 0.66 |
| fire severity + fire event + TPI + fire event x fire severity x TPI | 39 | 259.7 | -416.8 | 21.58 | 0.00 | 0.37 | 0.89 |
| pyrodiversity + elevation + pyrodiversity x elevation | 7 | 215.3 | -415.9 | 22.41 | 0.00 | 0.07 | 0.63 |
| pyrodiversity + fire severity + fire event + fire severity x pyrodiversity x fire event | 39 | 259.1 | -415.7 | 22.60 | 0.00 | 0.35 | 0.91 |
| null | 4 | 210.7 | -413.2 | 25.12 | 0.00 | 0.00 | 0.66 |
| pyrodiversity + dominant vegetation type + pyrodiversity x dominant vegetation type | 11 | 218.0 | -412.3 | 26.04 | 0.00 | 0.11 | 0.64 |
| pyrodiversity | 5 | 211.3 | -412.2 | 26.10 | 0.00 | 0.01 | 0.62 |
| fire severity + TPI + fire severity x TPI | 15 | 222.7 | -412.1 | 26.18 | 0.00 | 0.17 | 0.65 |
| fire severity + solar radiation + fire severity x solar radiation | 15 | 222.3 | -411.5 | 26.84 | 0.00 | 0.15 | 0.66 |
| pyrodiversity + solar radiation + pyrodiversity x solar radiation | 7 | 211.3 | -407.9 | 30.38 | 0.00 | 0.01 | 0.62 |
| fire severity + elevation + fire severity x elevation | 15 | 220.5 | -407.9 | 30.47 | 0.00 | 0.12 | 0.67 |
| fire severity + fire event + elevation + fire event x fire severity x elevation | 39 | 254.9 | -407.3 | 31.07 | 0.00 | 0.33 | 0.89 |
| pyrodiversity + fire severity + fire severity x pyrodiversity | 15 | 220.1 | -407.0 | 31.31 | 0.00 | 0.14 | 0.58 |
| fire severity + dominant vegetation type + fire severity x dominant vegetation type | 24 | 228.5 | -400.5 | 37.86 | 0.00 | 0.22 | 0.73 |
| fire severity + fire event + TPI + fire event x fire severity x TPI | 39 | 251.2 | -399.9 | 38.43 | 0.00 | 0.32 | 0.88 |
| fire severity + fire event + dominant vegetation type + fire event x fire severity x dominant vegetation type | 50 | 264.8 | -385.7 | 52.68 | 0.00 | 0.39 | 0.92 |
| (c) Relative phylogenetic diversity (RPD) | | | |  |  |  |  |
| fire severity + solar radiation + fire severity x solar radiation | 15 | 1.5 | 30.3 | 0.00 | 0.98 | 0.25 | 0.56 |
| pyrodiversity + fire event + TPI + fire event x pyrodiversity x TPI | 15 | -2.5 | 38.2 | 7.90 | 0.02 | 0.23 | 0.55 |
| pyrodiversity + fire severity + fire severity x pyrodiversity | 15 | -6.6 | 46.3 | 16.03 | 0.00 | 0.21 | 0.55 |
| fire severity + fire event + fire event x fire severity | 21 | 0.7 | 47.1 | 16.79 | 0.00 | 0.26 | 0.60 |
| burn severity | 9 | -14.5 | 48.2 | 17.89 | 0.00 | 0.14 | 0.46 |
| fire severity + TPI + fire severity x TPI | 15 | -11.3 | 55.8 | 25.54 | 0.00 | 0.17 | 0.46 |
| pyrodiversity + solar radiation + pyrodiversity x solar radiation | 7 | -21.0 | 56.8 | 26.54 | 0.00 | 0.08 | 0.35 |
| fire severity + elevation + fire severity x elevation | 15 | -11.9 | 57.0 | 26.77 | 0.00 | 0.16 | 0.48 |
| fire severity + fire event + TPI + fire event x fire severity x TPI | 39 | 22.6 | 57.4 | 27.12 | 0.00 | 0.39 | 0.66 |
| pyrodiversity + fire event + fire event x pyrodiversity | 9 | -20.4 | 59.9 | 29.66 | 0.00 | 0.07 | 0.40 |
| null | 4 | -27.0 | 62.3 | 32.01 | 0.00 | 0.00 | 0.30 |
| pyrodiversity | 5 | -26.5 | 63.4 | 33.18 | 0.00 | 0.01 | 0.32 |
| pyrodiversity + TPI + pyrodiversity x TPI | 7 | -25.0 | 64.7 | 34.46 | 0.00 | 0.03 | 0.33 |
| pyrodiversity + fire event + elevation + fire event x pyrodiversity x elevation | 15 | -16.0 | 65.2 | 34.93 | 0.00 | 0.12 | 0.47 |
| pyrodiversity + elevation + pyrodiversity x elevation | 7 | -25.4 | 65.4 | 35.19 | 0.00 | 0.03 | 0.32 |
| pyrodiversity + dominant vegetation type + pyrodiversity x dominant vegetation type | 11 | -21.0 | 65.7 | 35.46 | 0.00 | 0.08 | 0.35 |
| pyrodiversity + fire event + TPI + fire event x pyrodiversity x TPI | 15 | -16.7 | 66.5 | 36.27 | 0.00 | 0.11 | 0.46 |
| fire severity + dominant vegetation type + fire severity x dominant vegetation type | 24 | -7.5 | 71.4 | 41.17 | 0.00 | 0.21 | 0.52 |
| pyrodiversity + fire event + dominant vegetation type + fire event x pyrodiversity x dominant vegetation type | 24 | -8.2 | 72.9 | 42.65 | 0.00 | 0.20 | 0.46 |
| pyrodiversity + fire severity + fire event + fire severity x pyrodiversity x fire event | 38 | 11.1 | 77.0 | 46.73 | 0.00 | 0.34 | 0.67 |
| fire severity + fire event + elevation + fire event x fire severity x elevation | 39 | 10.4 | 81.8 | 51.57 | 0.00 | 0.33 | 0.66 |
| fire severity + fire event + TPI + fire event x fire severity x TPI | 39 | 8.8 | 84.9 | 54.63 | 0.00 | 0.33 | 0.65 |
| fire severity + fire event + dominant vegetation type + fire event x fire severity x dominant vegetation type | 50 | 18.7 | 106.5 | 76.28 | 0.00 | 0.40 | 0.64 |
| (d) Weighted endemism (WE) | | |  |  |  |  |  |
| pyrodiversity + TPI + pyrodiversity x TPI | 7 | -250.3 | 515.2 | 0.00 | 0.45 | 0.12 | 0.35 |
| pyrodiversity + fire event + TPI + fire event x pyrodiversity x TPI | 15 | -241.4 | 516.0 | 0.82 | 0.30 | 0.24 | 0.42 |
| pyrodiversity + elevation + pyrodiversity x elevation | 7 | -251.5 | 517.6 | 2.43 | 0.13 | 0.12 | 0.33 |
| pyrodiversity + fire event + fire event x pyrodiversity | 9 | -249.9 | 519.0 | 3.82 | 0.07 | 0.11 | 0.43 |
| fire severity + fire event + fire event x fire severity | 21 | -236.5 | 521.4 | 6.20 | 0.02 | 0.33 | 0.39 |
| pyrodiversity + fire event + elevation + fire event x pyrodiversity x elevation | 15 | -244.4 | 521.9 | 6.68 | 0.02 | 0.21 | 0.39 |
| pyrodiversity | 5 | -256.5 | 523.3 | 8.08 | 0.01 | 0.03 | 0.35 |
| null | 4 | -257.8 | 523.8 | 8.61 | 0.01 | 0.00 | 0.42 |
| burn severity | 9 | -253.2 | 525.6 | 10.35 | 0.00 | 0.07 | 0.44 |
| fire severity + solar radiation + fire severity x solar radiation | 15 | -246.4 | 526.0 | 10.80 | 0.00 | 0.16 | 0.57 |
| pyrodiversity + solar radiation + pyrodiversity x solar radiation | 7 | -256.4 | 527.6 | 12.39 | 0.00 | 0.03 | 0.34 |
| pyrodiversity + dominant vegetation type + pyrodiversity x dominant vegetation type | 11 | -252.6 | 528.9 | 13.66 | 0.00 | 0.10 | 0.34 |
| pyrodiversity + fire event + TPI + fire event x pyrodiversity x TPI | 15 | -248.1 | 529.4 | 14.15 | 0.00 | 0.12 | 0.50 |
| fire severity + TPI + fire severity x TPI | 15 | -248.8 | 530.9 | 15.66 | 0.00 | 0.14 | 0.41 |
| fire severity + elevation + fire severity x elevation | 15 | -249.2 | 531.6 | 16.38 | 0.00 | 0.13 | 0.43 |
| pyrodiversity + fire severity + fire severity x pyrodiversity | 15 | -250.3 | 533.7 | 18.51 | 0.00 | 0.12 | 0.37 |
| fire severity + fire event + TPI + fire event x fire severity x TPI | 39 | -220.9 | 544.3 | 29.07 | 0.00 | 0.46 | 0.63 |
| pyrodiversity + fire event + dominant vegetation type + fire event x pyrodiversity x dominant vegetation type | 24 | -244.7 | 545.9 | 30.67 | 0.00 | 0.20 | 0.42 |
| fire severity + dominant vegetation type + fire severity x dominant vegetation type | 24 | -246.1 | 548.7 | 33.53 | 0.00 | 0.16 | 0.53 |
| (e) Phylogenetic endemism (PE) | | |  |  |  |  |  |
| pyrodiversity + fire event + TPI + fire event x pyrodiversity x TPI | 15 | 737.9 | -1442.6 | 0.00 | 0.69 | 0.19 | 0.70 |
| pyrodiversity + fire event + fire event x pyrodiversity | 9 | 729.4 | -1439.7 | 2.83 | 0.17 | 0.10 | 0.65 |
| pyrodiversity + TPI + pyrodiversity x TPI | 7 | 726.4 | -1438.2 | 4.39 | 0.08 | 0.10 | 0.42 |
| fire severity + fire event + fire event x fire severity | 21 | 742.2 | -1435.9 | 6.61 | 0.03 | 0.22 | 0.75 |
| burn severity | 9 | 727.5 | -1435.9 | 6.62 | 0.03 | 0.10 | 0.50 |
| fire severity + TPI + fire severity x TPI | 15 | 733.5 | -1433.8 | 8.71 | 0.01 | 0.18 | 0.52 |
| pyrodiversity + fire event + elevation + fire event x pyrodiversity x elevation | 15 | 732.0 | -1430.8 | 11.77 | 0.00 | 0.13 | 0.69 |
| pyrodiversity + elevation + pyrodiversity x elevation | 7 | 721.8 | -1429.0 | 13.58 | 0.00 | 0.05 | 0.37 |
| null | 4 | 718.3 | -1428.3 | 14.20 | 0.00 | 0.00 | 0.34 |
| fire severity + elevation + fire severity x elevation | 15 | 730.4 | -1427.6 | 14.92 | 0.00 | 0.13 | 0.53 |
| fire severity + fire event + TPI + fire event x fire severity x TPI | 39 | 761.7 | -1420.8 | 21.74 | 0.00 | 0.33 | 0.87 |
| pyrodiversity + fire severity + fire severity x pyrodiversity | 15 | 726.2 | -1419.2 | 23.31 | 0.00 | NA | NA |
| pyrodiversity + fire severity + fire event + fire severity x pyrodiversity x fire event | 38 | 758.9 | -1418.7 | 23.81 | 0.00 | 0.34 | 0.84 |
| pyrodiversity + fire event + dominant vegetation type + fire event x pyrodiversity x dominant vegetation type | 24 | 737.1 | -1417.8 | 24.78 | 0.00 | 0.16 | 0.70 |
| fire severity + fire event + elevation + fire event x fire severity x elevation | 39 | 755.3 | -1408.0 | 34.54 | 0.00 | 0.30 | 0.77 |
| fire severity + fire event + TPI + fire event x fire severity x TPI | 39 | 752.9 | -1403.3 | 39.25 | 0.00 | 0.31 | 0.78 |
| fire severity + fire event + dominant vegetation type + fire event x fire severity x dominant vegetation type | 50 | 760.0 | -1376.1 | 66.46 | 0.00 | 0.35 | 0.86 |
| (f) Taxonomic beta diversity (TBD) | | |  |  |  |  |  |
| pyrodiversity + fire event + TPI + fire event x pyrodiversity x TPI | 15 | 183.6 | -334.1 | 0.00 | 0.97 | 0.42 | 0.91 |
| pyrodiversity + fire event + elevation + fire event x pyrodiversity x elevation | 15 | 179.8 | -326.5 | 7.58 | 0.02 | 0.34 | 0.92 |
| pyrodiversity + fire event + dominant vegetation type + fire event x pyrodiversity x dominant vegetation type | 24 | 190.6 | -324.7 | 9.40 | 0.01 | 0.58 | 0.88 |
| pyrodiversity + TPI + pyrodiversity x TPI | 7 | 167.9 | -321.0 | 13.05 | 0.00 | 0.51 | 0.64 |
| pyrodiversity + fire event + fire event x pyrodiversity | 9 | 168.7 | -318.4 | 15.75 | 0.00 | 0.21 | 0.92 |
| pyrodiversity + elevation + pyrodiversity x elevation | 7 | 164.1 | -313.6 | 20.54 | 0.00 | 0.41 | 0.63 |
| pyrodiversity + fire event + TPI + fire event x pyrodiversity x TPI | 15 | 173.0 | -312.7 | 21.35 | 0.00 | 0.26 | 0.93 |
| pyrodiversity + dominant vegetation type + pyrodiversity x dominant vegetation type | 11 | 165.5 | -307.3 | 26.81 | 0.00 | 0.47 | 0.61 |
| fire severity + fire event + fire event x fire severity | 21 | 177.6 | -306.8 | 27.29 | 0.00 | 0.26 | 0.94 |
| fire severity + fire event + elevation + fire event x fire severity x elevation | 39 | 204.5 | -306.5 | 27.59 | 0.00 | 0.40 | 0.98 |
| pyrodiversity + solar radiation + pyrodiversity x solar radiation | 7 | 159.5 | -304.2 | 29.89 | 0.00 | 0.30 | 0.60 |
| pyrodiversity | 5 | 155.7 | -301.0 | 33.05 | 0.00 | 0.18 | 0.59 |
| fire severity + fire event + TPI + fire event x fire severity x TPI | 39 | 201.8 | -301.0 | 33.13 | 0.00 | 0.43 | 0.96 |
| burn severity | 9 | 158.9 | -298.7 | 35.35 | 0.00 | 0.22 | 0.72 |
| null | 4 | 152.3 | -296.4 | 37.72 | 0.00 | 0.10 | 0.12 |
| pyrodiversity + fire severity + fire severity x pyrodiversity | 15 | 163.1 | -293.0 | 41.13 | 0.00 | 0.25 | 0.83 |
| fire severity + TPI + fire severity x TPI | 15 | 162.8 | -292.4 | 41.65 | 0.00 | 0.31 | 0.75 |
| fire severity + elevation + fire severity x elevation | 15 | 161.4 | -289.7 | 44.41 | 0.00 | 0.28 | 0.74 |
| fire severity + solar radiation + fire severity x solar radiation | 15 | 161.4 | -289.6 | 44.53 | 0.00 | 0.29 | 0.71 |
| fire severity + dominant vegetation type + fire severity x dominant vegetation type | 24 | 170.4 | -284.4 | 49.72 | 0.00 | 0.54 | 0.68 |
| pyrodiversity + fire severity + fire event + fire severity x pyrodiversity x fire event | 39 | 189.2 | -275.8 | 58.30 | 0.00 | 0.29 | 0.97 |
| fire severity + fire event + TPI + fire event x fire severity x TPI | 39 | 185.6 | -268.7 | 65.38 | 0.00 | 0.31 | 0.96 |
| fire severity + fire event + dominant vegetation type + fire event x fire severity x dominant vegetation type | 50 | 205.2 | -266.5 | 67.58 | 0.00 | 0.61 | 0.93 |
| (g) Phylogenetic beta diversity (PBD) | | |  |  |  |  |  |
| fire severity + dominant vegetation type + fire severity x dominant vegetation type | 24 | 220.5 | -384.5 | 0.00 | 0.48 | 0.48 | 0.65 |
| pyrodiversity + dominant vegetation type + pyrodiversity x dominant vegetation type | 11 | 204.1 | -384.5 | 0.03 | 0.47 | 0.36 | 0.54 |
| pyrodiversity + fire event + dominant vegetation type + fire event x pyrodiversity x dominant vegetation type | 24 | 218.1 | -379.8 | 4.71 | 0.05 | 0.48 | 0.68 |
| pyrodiversity + fire event + fire event x pyrodiversity | 9 | 197.7 | -376.3 | 8.17 | 0.01 | 0.28 | 0.45 |
| burn severity | 9 | 195.2 | -371.2 | 13.35 | 0.00 | 0.24 | 0.45 |
| null | 4 | 189.6 | -371.0 | 13.51 | 0.00 | 0.16 | 0.36 |
| pyrodiversity + fire event + TPI + fire event x pyrodiversity x TPI | 15 | 201.6 | -370.1 | 14.42 | 0.00 | 0.32 | 0.53 |
| pyrodiversity + TPI + pyrodiversity x TPI | 7 | 192.3 | -369.8 | 14.69 | 0.00 | 0.20 | 0.41 |
| pyrodiversity + elevation + pyrodiversity x elevation | 7 | 192.2 | -369.8 | 14.73 | 0.00 | 0.19 | 0.44 |
| pyrodiversity | 5 | 190.0 | -369.7 | 14.84 | 0.00 | 0.16 | 0.42 |
| pyrodiversity + solar radiation + pyrodiversity x solar radiation | 7 | 191.4 | -368.2 | 16.34 | 0.00 | 0.18 | 0.42 |
| pyrodiversity + fire event + TPI + fire event x pyrodiversity x TPI | 15 | 200.6 | -368.1 | 16.41 | 0.00 | 0.31 | 0.51 |
| pyrodiversity + fire event + elevation + fire event x pyrodiversity x elevation | 15 | 200.3 | -367.5 | 17.04 | 0.00 | 0.31 | 0.51 |
| fire severity + solar radiation + fire severity x solar radiation | 15 | 199.7 | -366.1 | 18.37 | 0.00 | 0.31 | 0.44 |
| fire severity + elevation + fire severity x elevation | 15 | 198.4 | -363.6 | 20.92 | 0.00 | 0.27 | 0.54 |
| fire severity + fire event + fire event x fire severity | 21 | 205.0 | -361.7 | 22.82 | 0.00 | 0.34 | 0.60 |
| fire severity + TPI + fire severity x TPI | 15 | 196.9 | -360.5 | 23.96 | 0.00 | 0.26 | 0.49 |
| fire severity + fire event + dominant vegetation type + fire event x fire severity x dominant vegetation type | 50 | 243.6 | -343.2 | 41.27 | 0.00 | 0.61 | 0.82 |
| fire severity + fire event + TPI + fire event x fire severity x TPI | 39 | 218.4 | -334.2 | 50.34 | 0.00 | 0.53 | 0.58 |
| fire severity + fire event + elevation + fire event x fire severity x elevation | 39 | 216.1 | -329.5 | 54.96 | 0.00 | 0.47 | 0.64 |
| fire severity + fire event + TPI + fire event x fire severity x TPI | 39 | 214.2 | -325.8 | 58.71 | 0.00 | 0.43 | 0.67 |
| pyrodiversity + fire severity + fire event + fire severity x pyrodiversity x fire event | 39 | 213.2 | -323.8 | 60.74 | 0.00 | 0.46 | 0.59 |

**Figures**

Figure S1: The (a) Venn diagram (overlap) in taxa (genera) between the Caples creek site (red) and the Long fire sites (blue). The distribution of plots in the two sites by (b) elevation, (c) topographic position, and (d) solar radiation.

Figure S2: Correlation between each of the paired taxonomic and phylogenetic based metrics including: (a) taxonomic richness (TR) and phylogenetic diversity (PD), (b) weighted endemism (WE) and phylogenetic endemism (PE), and (c) taxonomic beta diversity (TBD) and phylogenetic diversity (PBD) (regional calculation). Pearson’s correlation values (r) are listed on the top left of each graph.

Figure S3: The spatial distribution of fire return interval (row 1), fire severity (row 2), patch size (row 3), and pyrodiversity (Functional dispersion incorporating the three fire regime metrics) for each sample period (columns), based on sub-watersheds (HUC12s).

Figure S4: Example of a Caples creek plot located on a ridge and South facing, from the fire exclusion sample period (a, sampled 2013) and after the Caples fire (b, sampled 2020)

Figure S5: Example of Caples creek plots located in canyon bottom with dense tree cover, from the fire exclusion sample period (a, c; sampled 2013) and after the Caples fire (b, d; sampled 2020).

Figure S6: Example of Caples creek plots, representative of all slope positions, and dense canopy cover, from the fire exclusion sample period (a; sampled 2013) and after the Caldor fire (b; sampled 2022).

Figure S7. Example of Long plots, representative of high elevation fir forest, that burned at low to moderate severity during the Long fire (a; sampled 2013) and during the Caldor fire (b; sampled 2022).

Figure S8: Gamma diversity (regional species richness) for each sample period: (gray) fire exclusion, (blue) Long fire, (yellow) Caples fire, and (orange) Caldor fire.

**References**

Brooks, M.E., Kristensen, K., Van Benthem, K.J., Magnusson, A., Berg, C.W., Nielsen, A., *et al.* (2017). glmmTMB balances speed and flexibility among packages for zero-inflated generalized linear mixed modeling. *The R Journal*, 9, 378–400.

Burnham, K.P. & Anderson, D.R. (2004). Multimodel inference: understanding AIC and BIC in model selection. *Sociological methods & research*, 33, 2261–304.

Dailey, S., Reiner, A. & Ewell, C. (2020). 2019 Caples Fire, Eldorado National Forest. First Order Fire Effects, Assessed November 2019. *USDA Forest Service, Report*.

De Reu, J., Bourgeois, J., Bats, M., Zwertvaegher, A., Gelorini, V., De Smedt, P., *et al.* (2013). Application of the topographic position index to heterogeneous landscapes. *Geomorphology*, 186, 39–49.

Gotelli, N.J. & Colwell, R.K. (2001). Quantifying biodiversity: procedures and pitfalls in the measurement and comparison of species richness. *Ecology Letters*, 4, 379–391.

Jepson Flora Project (eds.). (2025). *Jepson eFlora*. Available at: https://ucjeps.berkeley.edu/eflora/. Last accessed 20 August 2025.

Laliberté, E. & Legendre, P. (2010). A distance‐based framework for measuring functional diversity from multiple traits. *Ecology*, 91, 299–305.

Missouri Botanical Garden. (2025). *Tropicos*. Available at: https://tropicos.org/name/13010071. Last accessed 20 August 2025.

Munz, P.A. & Keck, D.D. (1959). *A California Flora*. University of California Press.

Oksanen, J., Simplson, G., Blanchet, F., Kindt, R., Legendre, P., Minchin, P., *et al.* (2025). vegan: Community Ecology Package. *R package version 2.6-10*.

Steel, Z.L., Collins, B.M., Sapsis, D.B. & Stephens, S.L. (2021). Quantifying pyrodiversity and its drivers. *Proceedings of the Royal Society B: Biological Sciences*, 288.

Steel 2021 Github: Pyrodiversity. https://github.com/zacksteel/pyrodiversity. [accessed January 20, 2026]

Thornhill, A.H., Baldwin, B.G., Freyman, W.A., Nosratinia, S., Kling, M.M., Morueta-Holme, N., *et al.* (2017). Spatial phylogenetics of the native California flora. *BMC Biol*, 15, 96.

Wilkin, K., Ponisio, L., Fry, D.L., Collins, B.M., Moody, T. & Stephens, S.L. (2021). Drivers of understory plant communities in Sierra Nevada mixed conifer forests with pyrodiversity. *fire ecol*, 17, 30.
